# Correlates and determinants of physical activity among older adults of lower versus higher socio-economic status: a systematic review and meta-analysis

**DOI:** 10.1186/s12966-025-01775-y

**Published:** 2025-06-23

**Authors:** Olivia S. Malkowski, Jessica Harvey, Nick P. Townsend, Mark J. Kelson, Charlie E. M. Foster, Max J. Western, Physical Activity Inequalities in Later Life consortium, Ahmad Alkhatib, Kyle J. Bourassa, Hannah Burnett, Mia Cajita, Simon Cook, Anne Corbett, Janie Corley, Simon R. Cox, Byron Creese, Katie Dudman, Joanne K. Garrett, Adam Hampshire, Coral L. Hanson, Melvyn Hillsdon, Jeroen Lakerveld, Agnieszka Lemanska, Laura Macdonald, Jane Maddock, Adilson Marques, Irene Mosca, David Ogilvie, Déborah Oliveira, Tytti P. Pasanen, Ilaria Pina, Alex V. Rowlands, Shaun Scholes, Lindsey G. Smith, Emma Solomon-Moore, Emmanuel Stamatakis, Yannick Stephan, Clare Stevinson, Tessa Strain, Cecilie Thøgersen-Ntoumani, Carri Westgarth

**Affiliations:** 1 Centre for Motivation and Behaviour Change, Department for Health, University of Bath, Bath, UK; 2 Centre for Exercise, Nutrition and Health Sciences, School for Policy Studies, University of Bristol, Bristol, UK; 3 Institute of Data Science and Artificial Intelligence, Department of Mathematics, University of Exeter, Exeter, UK

**Keywords:** Older adults, Physical activity, Systematic review, Meta-analysis, Correlates, Health inequalities, Socio-economic status

## Abstract

**Background:**

Understanding socio-economic differences in the factors influencing physical activity among older adults is essential for developing comprehensive interventions. We aimed to quantify the associations of modifiable correlates and determinants on physical activity among older adults of lower versus higher socio-economic status in the United Kingdom.

**Methods:**

In this systematic review and meta-analysis, we searched MEDLINE, Embase, Web of Science, Cochrane Central Register of Controlled Trials (CENTRAL), and Scopus from inception to December 2023, for peer-reviewed studies published in English, investigating associations between a modifiable factor as an independent variable and physical activity as a dependent variable, by socio-economic status (defined according to various area- and individual-level indicators, including neighbourhood deprivation, wealth or income, education, and occupational class), in samples of community-dwelling older adults aged 60+ years in the United Kingdom. Hospitalised and institutionalised populations were excluded. Random effects meta-analyses were performed separately for people of lower and higher socio-economic status. Risk of bias was assessed with the Mixed Methods Appraisal Tool. This study was registered with the International prospective register of systematic reviews (PROSPERO; CRD42022351708).

**Results:**

Searches identified 11,472 references; seventy-seven studies met the selection criteria, of which fifty-one contributed to meta-analyses (N range = 134–29,280). Of the exposures positively associated with physical activity, physical function, social participation, and perception of general health had the largest effect sizes (standardised mean difference [SMD] range = 0.53–0.81; I^2^ range = 54.81–91.00%). Estimates were comparable among older adults of lower and higher socio-economic status, except for the presence of built physical activity facilities, access to walking and cycling infrastructure, and less smoking, which were positively associated with physical activity only among individuals of lower socio-economic status.

**Conclusions:**

Our results suggest researchers need to better understand discrepancies in the prevalence of the assessed correlates (e.g., fewer participants of lower socio-economic status reported good physical function) to inform policies that reduce inequalities in older adults’ physical activity levels. However, most studies were cross-sectional. Future longitudinal and experimental research should gauge the suitability of these correlates as intervention targets.

**Supplementary Information:**

The online version contains supplementary material available at 10.1186/s12966-025-01775-y.

## Background

Older adults are the fastest growing segment of Western societies. As populations age, finding ways of extending the span of healthy life expectancy is imperative. The importance of health inequalities becomes apparent when we consider that people of lower socio-economic status (SES), defined according to various area-level (e.g., neighbourhood deprivation) or individual-level (e.g., wealth or income, education, occupational class) indicators, have, on average, poorer health outcomes, including higher disease prevalence and reduced quality of life [1]. Addressing social inequalities within countries would not only benefit the economy, but more importantly, ensure a fair distribution of health and wellbeing [1]. Of note, many of the key behavioural risk factors for ill health, including lack of physical activity, follow the social gradient; tackling health inequalities therefore requires a focus on health behaviours [1].

Regular physical activity, defined broadly as “any bodily movement produced by skeletal muscles that requires energy expenditure” [2], is associated with a range of psychosocial and health-related benefits [3]. Nevertheless, fewer than 25% of older adults aged 60+ years in the United Kingdom (UK) comply with recommended guidelines of 150 min of moderate-intensity or 75 min of vigorous-intensity physical activity, and two or more days of muscle-strengthening activity per week [4]. Moreover, people of lower SES engage in less physical activity relative to those of higher SES [5], with the gap widening up until the age of 85 years [6]. From a public health perspective, it is paramount to devote special attention towards uncovering the mechanisms underlying this divide.

Most interventions targeting improvements in physical activity have been evaluated in the general older adult population with little regard for their impact on social inequalities [7]. Furthermore, systematic reviews on randomised controlled trials of physical activity interventions have found weak or inconclusive effects among individuals of lower SES [8, 9], suggesting a lack of specificity and relevance to those from less advantaged backgrounds. To increase their chances of success, interventions should focus on the most influential correlates (factors associated with physical activity albeit not necessarily causally so) or determinants (factors identified in aetiological study designs) of physical activity [10].

Behavioural theories and models frequently guide the selection of variables in empirical studies. Over recent decades, research on correlates and determinants of physical activity has progressed beyond addressing personal factors (e.g., biological, psychological, and behavioural) in isolation and towards considering social, environmental, and policy factors synchronously. The socio-ecological model of health is one such framework that emphasises the multiple layers of influence upon human behaviour and their interrelationships [11]. Through this lens, modifiable factors are characteristics susceptible to change through public policies, the physical or social environment, and individuals’ own choices or efforts.

To date, several systematic reviews have examined socio-economic disparities in physical activity participation [12, 13], and correlates or determinants of physical activity among older adults [14, 15]. Consistent with a socio-ecological approach, these reviews have identified factors across the intrapersonal, interpersonal, environmental, and public policy levels. However, to our knowledge, no reviews have examined whether socio-economic differences in the correlates or determinants of physical activity exist among older adults or reported pooled analyses in a way that separates the associations of correlates or determinants with physical activity behaviour in lower and higher socio-economic groups. A meta-analysis on this topic is therefore a key first step towards developing multilevel lifestyle interventions and updating policy on physical activity and ageing. Importantly, correlates and determinants of physical activity vary between countries; hence, the UK is required to identify and implement a strategic combination of policy actions according to their national context [16].

The aim of this systematic review was to comprehensively synthesise the literature on, and explore statistical associations of, modifiable correlates and determinants on physical activity behaviour among UK-based older adults of lower versus higher SES. While we recognise that SES operates on a continuum, socio-economic indicators are frequently collapsed into dichotomous variables in the literature, such as receiving no education or some formal primary/secondary education versus completing tertiary education or belonging to the bottom quintiles of a wealth/income distribution versus everyone else. For comparability across studies and to maximise the use of available data for inclusion in this review, older adults were classified into low versus medium/high SES groups.

## Methods

### Search strategy and selection criteria

This systematic review with meta-analysis was restricted to original, peer-reviewed studies. Articles were eligible if they (a) involved community-dwelling UK older adults aged 60+ years; (b) investigated associations between a modifiable socio-ecological (intrapersonal, interpersonal, environmental, public policy) factor as an independent variable and physical activity as a dependent variable; and (c) included one socio-economic sub-group (e.g., all lower-SES participants) or reported results by SES. Examples of non-modifiable and/or socio-demographic variables that were not considered as potential correlates of physical activity behaviour were: any measure or proxy (e.g., housing tenure, access to a car, number of vehicles owned) of SES, as these variables were used to stratify analyses; demographic variables (e.g., age, biological sex, ethnicity, race, area of residence, living status, number of children, marital status, religious affiliation, political affiliation), as we originally planned to include some of these participant characteristics (if available) as fixed effects in the meta-analyses; diagnosed physical or mental health conditions/disabilities, as these variables were frequently used to characterise the participant sample of a given study rather than treated as modifiable correlates of physical activity (we did however consider anxiety or depressive symptoms, body mass index, and perception of general health as modifiable psychological or lifestyle correlates of physical activity); and previous physical activity (as well as any other variables captured before older age). We also included articles that summarised physical activity barriers, enablers, and motives in community-dwelling UK older adults, descriptively by SES, although recognise these are usually self-reported after an event (i.e., physical activity), whereas correlates and determinants should be associated with participation in a temporal sequence. For studies where it was possible to extract data by age, country, and SES from published articles, or separate information was obtainable by contacting the study authors or through OSM accessing public datasets, only participants who met the eligibility criteria were included. Where separate information was not obtainable, we included the study if the mean age of participants was ≥ 60 years and no participants were aged less than 50 years, contingent on all other criteria being met. Articles were excluded if they (a) focused exclusively on hospitalised or institutionalised populations; or (b) were not published in English. Conference abstracts, dissertations and theses, editorials, opinions, letters, trial protocols, reviews, and case reports were not considered.

Systematic searches were performed by OSM in five electronic databases from database origin to July 25, 2022; and updated on April 24, 2023: MEDLINE (PubMed interface), Embase (Embase.com interface), Web of Science, Cochrane Central Register of Controlled Trials (CENTRAL), and Scopus. The search strategy is available in Additional file 1. There were no restrictions on publication date. Additionally, trial registries (International Standard Randomised Controlled Trial Number [ISRCTN] registry; ClinicalTrials.gov) and the Open Science Framework were searched for relevant ongoing and unpublished studies by OSM from inception to June 16, 2023. The reference lists of published reviews (retrieved by searching MEDLINE from inception to June 16, 2023) were manually scanned by OSM to identify other eligible studies. A final update of the main search across the five electronic databases was conducted by OSM on December 18, 2023. The reference lists of all included studies were manually scanned by OSM to identify other potentially eligible studies that were not uncovered by the systematic searches.

Results were imported into EndNote [17], and Covidence systematic review software [18], where duplicates were automatically removed. Additional duplicates were identified in EndNote following Bramer and colleagues’ method [19]. One reviewer (OSM) independently screened all results against the selection criteria, in two stages: (1) titles and abstracts; and (2) full articles. At each stage, 30% of results were independently double screened (over 90% proportionate agreement achieved) by two other reviewers (MJW and JH; 15% each based on the original search results). A pilot trial, where reviewers involved in screening decided whether to include or exclude a given result, was carried out at the title and abstract stage. Discrepancies at the title and abstract stage were resolved through discussion between the two reviewers concerned; conflicts at the full text stage were discussed and resolved with a fourth reviewer (NPT). Single-screened articles were then re-visited by OSM to ensure consistency in approach.

Data extraction was undertaken by OSM; two additional reviewers (MJW and JH) each independently validated a 15% random sample of included papers. Sample characteristics, methods (e.g., study design, theoretical frameworks, measures, follow-up, study-level controls), results (e.g., effect sizes, attrition rate), limitations, and general information (e.g., ethical approval, funding sources, and possible conflicts of interest) were extracted into a standard form (see Additional file 2). Where relevant, we contacted authors for supplementary information (e.g., summary statistics, individual participant data where permitted, and/or syntax files for public datasets). If authors did not respond or were unable to provide additional summary statistics, or it was appropriate for OSM to perform supplementary analyses directly and the dataset underlying the study findings was openly available, OSM accessed the data, attempted to replicate the sample from the original publication based on the study methods, and if successful, limited the sample to participants eligible for the review, and extracted the necessary data. We created a hierarchy of preferred metrics for SES (1. specific measure of SES or index of deprivation; 2. wealth or income; 3. education; and 4. occupational class). This hierarchy was informed by a previous review [9] and chosen on the grounds that specific measures of SES or composite indices of deprivation capture more complex or multidimensional domains of SES than individual components alone. As a contemporary marker of SES, wealth or income is also considered a more appropriate measure for older adults relative to education and occupational class, which are often determined in early adulthood [20]. Definitions of low and medium/high SES were decided on a study-by-study basis. For indices of deprivation, and wealth or income, quartile, quintile, or decile cut-points were most frequently used. Occasionally, it was necessary to decide on specific thresholds (e.g., below versus above or equal to £20,000) for dichotomising wealth or income; these cut-points were implemented as consistently as possible across studies and adapted as required based on the available measures (e.g., whether continuous or categorical scales were used). For education, most authors used a split between primary/secondary education and tertiary education (i.e., degree or equivalent). Occupational or social class was predominantly categorised as manual versus non-manual. One study used housing tenure (i.e., rented versus owned) as a proxy for SES. Additional file 5 specifies the socio-economic indicators and definitions employed for each included study. Where multiple physical activity outcome measures were available, we selected the one included in statistical analyses with the exposure(s); or extracted data in line with the following hierarchy of preferred metrics: (1) any measure of moderate-to-vigorous physical activity; and (2) the most generalisable measure in the study (e.g., overall/leisure-time physical activity prioritised over domain-specific/occupational physical activity). If the preferred physical activity outcome measure was assessed using both self-report and device-based instruments in a study, the device-based measure was prioritised. For longitudinal studies, outcome data were extracted according to the waves of data collection in the statistical analyses for the variables of interest; where there was a choice of more than one follow-up wave, the earliest wave was chosen to minimise attrition.

The protocol, which included a concurrent review of qualitative evidence (reported separately), was registered with the International prospective register of systematic reviews (PROSPERO; CRD42022351708) and approved by the Research Ethics Approval Committee for Health [EP 23 010] at the University of Bath. The Preferred Reporting Items for Systematic Reviews and Meta-Analyses (PRISMA) guidelines were followed (see Additional file 3) [21].

### Data analysis

Independent variables were reviewed for similarities and allocated into exposure categories. Where multiple variables from the same study represented an exposure, the one most aligned with measures from the other studies was selected. If data from multiple studies were available from the same dataset, and for the same exposure, the following decision list dictated which study to incorporate in the meta-analysis: (1) recency (more recent data collection waves prioritised); (2) study design (longitudinal associations prioritised); (3) generalisability (broader inclusion criteria prioritised); and (4) physical activity outcome measure (device-based measures prioritised). This applied to articles reporting data on a duplicate exposure and one or more unique exposures from the same dataset as other studies prioritised according to this hierarchy (studies only reporting data on a duplicate exposure from the same dataset as other studies prioritised according to this hierarchy were excluded at the full text screening stage).

If three or more studies had an equivalent exposure, inverse-variance random effects meta-analyses were performed independently for participants of lower versus higher SES. Where physical activity was reported as a continuous outcome for some studies and a dichotomous outcome for others, effect sizes were re-expressed as standardised mean differences (SMDs) with 95% confidence intervals (CI). Higher scores reflected better outcomes (e.g., greater volume/level/intensity of physical activity). In studies with dichotomous outcomes, 0.5 was added to each cell of those 2×2 tables with at least one cell equal to zero. Events reflected better outcomes (e.g., meeting physical activity guidelines). Analyses were replicated with odds ratios for comparison, although SMDs are prioritised in the text. In cases where both continuous and dichotomous outcome data were available, selections were based on the first chronological (or “primary”) operationalisation of physical activity in the published paper. As effect sizes were predominantly calculated from summary statistics, no exposure had adjusted estimates available for three or more studies, or complete participant characteristics for the samples in the review. Therefore, in contrast to our protocol, unadjusted estimates were used for the main analyses, and these did not control for fixed effects, such as biological sex.

In addition to meta-analyses combining continuous and dichotomous outcomes, separate models were run for each type of outcome data: (1) for studies presenting continuous outcomes using SMDs; and (2) for studies presenting dichotomous outcomes using relative risks and odds ratios. Studies presenting continuous *and* dichotomous outcomes were included in both separate models for the exposure. Up to four sensitivity or sub-group analyses were performed per exposure for each of the combined and separate meta-analyses. First, models were repeated discarding change scores. Then, if at least three studies were available for each comparator, sub-group analyses were performed by (a) SES measure (if at least three studies were available for two or more SES indicators); (b) study design (i.e., cross-sectional versus longitudinal); and (c) outcome measure (i.e., self-report versus device-based). In contrast to our protocol, sub-group differences between randomised controlled trials and other quantitative designs were not examined, as virtually all studies were observational.

The Capability, Opportunity, Motivation, and Behaviour (COM-B) model was used as an analytical framework to structure the results; this model identifies physical (e.g., physical strength and mobility) and psychological (e.g., knowledge and psychological skills) *capability*, physical (e.g., environmental context and resources) and social (e.g., social networks and cultural norms) *opportunity*, and reflective (e.g., planning and decision-making) and automatic (e.g., emotional responses and impulses) *motivation* as three essential conditions for behaviours like physical activity to occur [22]. The COM-B model accommodates intrapersonal, interpersonal, environmental, and public policy variables, but unlike the socio-ecological model, is specific to behaviour change. As such, we deviated from our protocol by drawing on the COM-B model rather than the socio-ecological model to organise our data, as this was deemed more appropriate for mapping the influence of the assessed correlates on older adults’ physical activity behaviour. An element of flexibility was, however, maintained to account for new variables and/or unexpected relationships. Notably, we identified a fourth component, termed *health*, with two sub-components: (1) general health and wellbeing; and (2) health behaviours, which are likely to interact with, or underpin, the other domains of the COM-B model. Indeed, the correlates related to general health and wellbeing exhibited some overlap with the other COM-B domains. Nonetheless, they could not definitively be attributed to a single domain (e.g., depressive symptoms could plausibly fit under capability and/or motivation). Furthermore, although health behaviours, such as fruit and vegetable consumption or sleep, are modifiable and worth investigating as correlates of physical activity, these variables did not align with any of the COM-B domains or sub-domains, reinforcing the utility of the newly coined *health* component.

The Mixed Methods Appraisal Tool (MMAT) was used to assess risk of bias based on the content of the original studies, rather than the sub-samples included in the meta-analyses [23]. OSM appraised all studies independently; MJW and JH each independently rated a 15% random sample, with over 80% proportionate agreement. Appraisals were compared, and conflicts resolved via discussion with NPT, with OSM using the reasoning from this process to verify the remaining papers. Modified indicators were developed to enable ratings to be applied consistently (see Additional file 4). As the MMAT does not compute an overall score from the ratings of each criterion, sensitivity analyses using only studies at “low risk of bias” were not conducted. It should be noted that, for pragmatic reasons, authors contacted for supplementary information were asked to share unadjusted bivariate study-level data, separately for lower and/or higher socio-economic sub-groups (created based on the preferred SES metric available in the study in accordance with our hierarchy as well as thresholds or categories decided by OSM), and of the datasets accessed and analysed by OSM, frequently only the unadjusted associations reported in the original studies could be replicated (and not the estimates from models adjusting for confounding variables). Accordingly, the risk of bias ratings applied to the original analyses conducted in the “parent studies” might not accurately reflect the risk of bias of the data contributing towards the meta-analyses when adjustments for confounding variables were removed. Self-reported physical activity measures were evaluated using criteria adapted from the Quality Assessment of Physical Activity Questionnaires (QAPAQ) checklist [24]. We deviated from our protocol by not using the Grading of Recommendations Assessment, Development and Evaluation (GRADE) approach to assess the certainty of evidence [25]. As our research question centred on socio-economic inequalities in the correlates or determinants of physical activity among older adults and, subsequently, analyses were often based on sub-samples of the original publications, assessing the certainty of evidence was not deemed appropriate or relevant. Instead, for each synthesis, we summarised risk of bias among the contributing studies.

The between-study variance (${\tau }^{2}$) was used to detect heterogeneity, in addition to the I^2^ statistic. If at least ten studies contributed to a meta-analysis, publication bias was assessed using funnel plots and the Egger regression asymmetry test [26]. Meta-analyses were performed in R version 4.2.3 with RStudio version 2021.09.1, using the *metafor* package [27]. *p* < 0.05 indicated statistical significance.

## Results

**Fig. 1:**
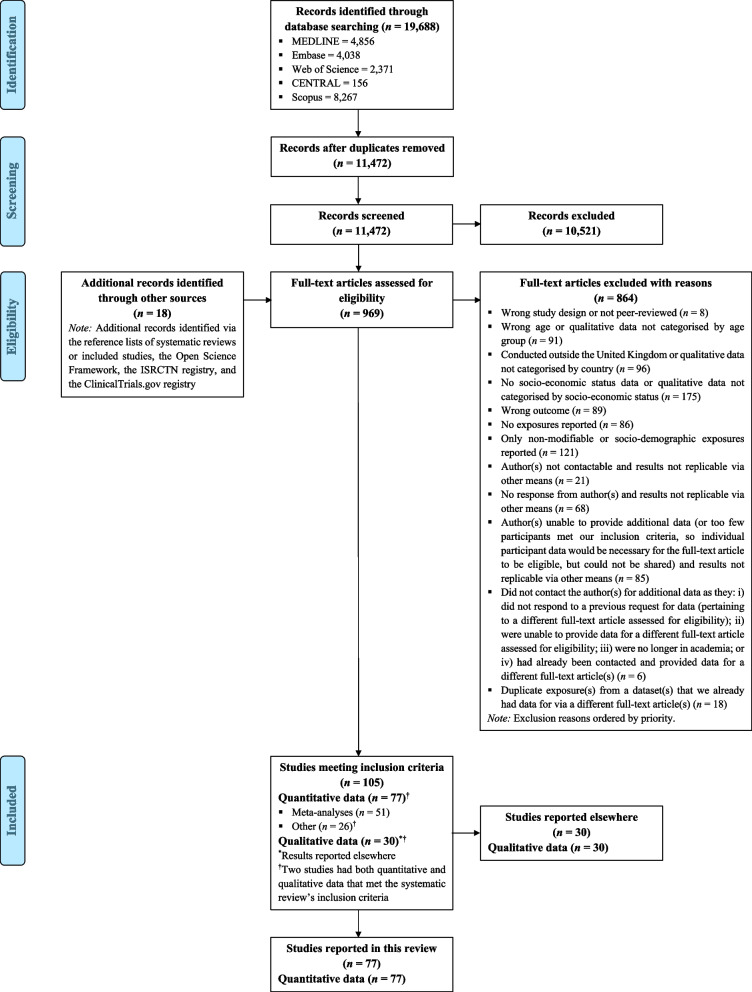
PRISMA flow diagram of the study selection process *Note:* CENTRAL, Cochrane Central Register of Controlled Trials; ISRCTN, International Standard Randomised Controlled Trial Number; PRISMA, Preferred Reporting Items for Systematic Reviews and Meta-Analyses

We screened 11,472 references and examined the full text of 969. Seventy-seven studies met the selection criteria (Fig. 1) [28–104]. A detailed overview of study characteristics, SES indicators (including how these were dichotomised), and physical activity measures is provided in Additional file 5. The best available measure of SES in each study was selected in accordance with our pre-specified priority list, with most participants being assigned to lower or higher SES sub-groups based on indicators of wealth or income (k = 28), followed by area deprivation (e.g., indices of multiple deprivation; k = 22), education (k = 14), occupational class (k = 12), and housing tenure (k = 1). Fifty-one studies contributed to at least one meta-analysis [28–78]. Sample sizes for the combined meta-analyses ranged from 134 to 29,280. The total sample size is not reported so as not to overcount participants from the same dataset or study who may have contributed to more than one meta-analysis (i.e., for different exposures). However, the sample sizes for each individual meta-analysis are presented. The way physical activity was measured and analysed varied widely between studies. The meta-analyses involved forty-five studies using self-reported measures and six studies using device-based measures of physical activity. The majority (k = 32) of studies reported dichotomous physical activity outcome data, thirteen reported continuous physical activity outcome data, and six reported dichotomous and continuous physical activity outcome data.

The twenty-six remaining studies descriptively summarised barriers, enablers, or motives by SES, reported on exposures assessed too infrequently to be meta-analysed, or produced effect sizes which could not be pooled with those of other studies [79–104]. Some examples of exposures that could not be meta-analysed were bodily aches or pain (physical capability), knowledge of the physical activity guidelines (psychological capability), land use (physical opportunity), social norms (social opportunity), personality traits (reflective motivation), the functioning of physiological systems (general health and wellbeing), and television viewing (health behaviours). Further details about the collection and analysis of the physical activity measures, as well as the descriptive barriers, enablers, motives, and exposures that could not be meta-analysed, can be found in the dataset located in the “Data” sub-folder of the GitHub repository (see Availability of data and materials).

Risk of bias assessments for all included studies are presented in Table 1, and risk of bias assessments by exposure-outcome categories for each combined meta-analysis are presented in Additional file 6.

**Table 1:** Risk of bias of the included studies

Methodological quality criteria for each category of studies	Responses		
	*Yes (%)*	*No (%)*	*Can’t tell (%)*
2. Quantitative randomised controlled trials (k = 2)			
2.1. Is randomisation appropriately performed?	100.0	0.0	0.0
2.2. Are the groups comparable at baseline?	100.0	0.0	0.0
2.3. Are there complete outcome data?	50.0	50.0	0.0
2.4. Are outcome assessors blinded to the intervention provided?	50.0	50.0	0.0
2.5. Did the participants adhere to the assigned intervention?	50.0	50.0	0.0
3. Quantitative non-randomised (k = 71)			
3.1. Are the participants representative of the target population?	29.6	70.4	0.0
3.2. Are measurements appropriate regarding both the outcome and intervention (or exposure)?	21.1	78.9	0.0
3.3. Are there complete outcome data?	74.6	16.9	8.5
3.4. Are the confounders accounted for in the design and analysis?	84.5	15.5	0.0
3.5. During the study period, is the intervention administered (or the exposure occurred) as intended?	18.3	81.7	0.0
4. Quantitative descriptive (k = 1)			
4.1. Is the sampling strategy relevant to address the research question?	100.0	0.0	0.0
4.2. Is the sample representative of the target population?	0.0	100.0	0.0
4.3. Are the measurements appropriate?	0.0	100.0	0.0
4.4. Is the risk of nonresponse bias low?	0.0	100.0	0.0
4.5. Is the statistical analysis appropriate to answer the research question?	100.0	0.0	0.0
5. Mixed methods (k = 3)			
5.1. Is there an adequate rationale for using a mixed methods design to address the research question?	100.0	0.0	0.0
5.2. Are the different components of the study effectively integrated to answer the research question?	100.0	0.0	0.0
5.3. Are the outputs of the integration of qualitative and quantitative components adequately interpreted?	66.7	33.3	0.0
5.4. Are divergences and inconsistencies between quantitative and qualitative results adequately addressed?	100.0	0.0	0.0
5.5. Do the different components of the study adhere to the quality criteria of each tradition of the methods involved?	0.0	100.0	0.0

Risk of bias was assessed using the Mixed Methods Appraisal Tool, Version 2018. k, number of studies. “Yes” rating: the criterion is met; “No” rating: the criterion is not met; “Can’t tell” rating: there is not enough information to judge if the criterion is met or not

**Fig. 2:**
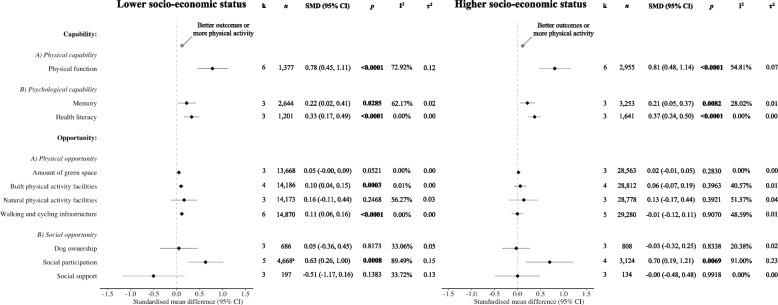
Associations of COM-B correlates with physical activity in older adults by SES *Note:* SMD, standardised mean difference; CI, confidence intervals; k, number of studies;* n*, number of participants; I^2^/τ^2^, heterogeneity statistics; SES, socio-economic status. As per our protocol, we did not include interaction terms between the respective exposures and SES in the meta-analytic models. Given that the original study authors were asked to create two sub-groups in their datasets according to SES (i.e., lower and higher) and provide unadjusted data for each of these two sub-groups, we conducted stratified meta-analyses instead. Random effects meta-analyses were performed separately for each exposure. ^a^The sample size from one contributing study was missing for the 60+ years sub-sample

Pooled analyses (Fig. 2) revealed that physical capability, represented by physical function (lower SES: SMD = 0.78, 95% CI = 0.45 to 1.11; higher SES: SMD = 0.81, 95% CI = 0.48 to 1.14), and psychological capability, the latter sub-divided into memory (lower SES: SMD = 0.22, 95% CI = 0.02 to 0.41; higher SES: SMD = 0.21, 95% CI = 0.05 to 0.37) and health literacy (lower SES: SMD = 0.33, 95% CI = 0.17 to 0.49; higher SES: SMD = 0.37, 95% CI = 0.24 to 0.50), were positively associated with physical activity in older adults of lower and higher SES. Regarding physical opportunity exposures, the amount of neighbourhood green space approached statistical significance in lower-SES older adults (SMD = 0.05, 95% CI = −0.00 to 0.09), but there was no evidence of an association in higher-SES older adults (SMD = 0.02, 95% CI = −0.01 to 0.05). There was a statistically significant positive association of built physical activity facilities only in lower-SES older adults (SMD = 0.10, 95% CI = 0.04 to 0.15). The presence of natural facilities was not significantly associated with physical activity in participants of lower or higher SES (*ps* ≥ 0.05). Analyses identified a statistically significant positive association of walking and cycling infrastructure in lower- (SMD = 0.11, 95% CI = 0.06 to 0.16), but not higher-SES (SMD = −0.01, 95% CI = −0.12 to 0.11), individuals. Among factors related to social opportunity, social participation, but not dog ownership or social support (*ps* ≥ 0.05), was positively associated with physical activity in both SES sub-groups (lower SES: SMD = 0.63, 95% CI = 0.26 to 1.00; higher SES: SMD = 0.70, 95% CI = 0.19 to 1.21). There were sufficient studies (k = 3) to conduct a meta-analysis on motivational exposures only among lower-SES older adults. No evidence of an association between reflective motivation and physical activity was found in this sub-group (SMD = 0.04, 95% CI = −0.33 to 0.40, *p* = 0.8507).

**Fig. 3:**
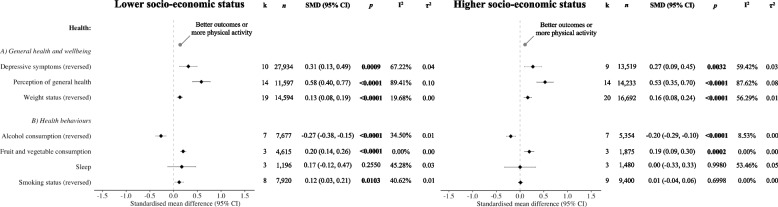
Associations of health-related correlates with physical activity in older adults by SES *Note:* SMD, standardised mean difference; CI, confidence intervals; k, number of studies;* n*, number of participants; I^2^/τ^2^, heterogeneity statistics; SES, socio-economic status. As per our protocol, we did not include interaction terms between the respective exposures and SES in the meta-analytic models. Given that the original study authors were asked to create two sub-groups in their datasets according to SES (i.e., lower and higher) and provide unadjusted data for each of these two sub-groups, we conducted stratified meta-analyses instead. Random effects meta-analyses were performed separately for each exposure

General health and wellbeing exposures (Fig. 3), including fewer depressive symptoms (lower SES: SMD = 0.31, 95% CI = 0.13 to 0.49; higher SES: SMD = 0.27, 95% CI = 0.09 to 0.45), better perceived general health (lower SES: SMD = 0.58, 95% CI = 0.40 to 0.77; higher SES: SMD = 0.53, 95% CI = 0.35 to 0.70), and lower body mass index (lower SES: SMD = 0.13, 95% CI = 0.08 to 0.19; higher SES: SMD = 0.16, 95% CI = 0.08 to 0.24), were positively associated with physical activity in lower- and higher-SES participants. In terms of health behaviours, lower alcohol intake was negatively associated (lower SES: SMD = −0.27, 95% CI = −0.38 to −0.15; higher SES: SMD = −0.20, 95% CI = −0.29 to −0.10), whereas higher fruit and vegetable consumption was positively associated (lower SES: SMD = 0.20, 95% CI = 0.14 to 0.26; higher SES: SMD = 0.19, 95% CI = 0.09 to 0.30), with physical activity in lower- and higher-SES sub-groups. There was no statistically significant association of sleep among individuals in either SES sub-group (*ps* ≥ 0.05). Less smoking was positively associated with physical activity only in lower-SES older adults (SMD = 0.12, 95% CI = 0.03 to 0.21). Funnel plots did not indicate publication bias for meta-analyses investigating associations of depressive symptoms, perception of general health, or weight status with physical activity (*ps* ≥ 0.05).

Patterns of association were broadly similar in sensitivity analyses (1) with odds ratios as “effect measures”, (2) omitting change scores, and (3) limited to continuous or dichotomous outcomes. The association of fewer depressive symptoms with physical activity was attenuated in studies using deprivation as a socio-economic indicator, while among higher-SES individuals, a positive association of lower weight status was identified when SES was defined by deprivation or income, but not occupational class. Minor discrepancies also emerged in sub-group analyses by study design (statistically significant positive association of lower weight status in cross-sectional but not longitudinal designs) and outcome measure (in lower-SES participants, there was a statistically significant association of weight status in studies using self-report but not device-based measures of physical activity). A post-hoc exploratory analysis of studies with data on both lower and higher socio-economic sub-groups compared the raw number of participants exposed versus unexposed to each assessed correlate, whereby *exposed* was defined as having better physical or cognitive function, more physical or social opportunities for physical activity, better health and wellbeing (e.g., fewer depressive symptoms, better perceived general health, or lower body mass index), and engagement in healthy lifestyle behaviours (e.g., lower alcohol consumption, higher fruit and vegetable consumption, better sleep, or less smoking). Information on how each continuous or categorical independent variable was split into exposed and unexposed groups, both for the meta-analyses and the post-hoc exploratory analysis, is provided in the dataset located in the “Data” sub-folder of the GitHub repository (see Availability of data and materials). This post-hoc analysis demonstrated that a greater proportion of higher-SES individuals reported good social participation (47.0% versus 28.6%) and perceived general health (72.0% versus 57.9%) relative to their lower-SES counterparts (Table 2).

**Table 2:** Prevalence of correlates in older adults of lower versus higher SES

Exposures	Lower socio-economic status *n* (%)	Higher socio-economic status *n* (%)	Association
Capability:			
A) *Physical capability*			
Physical function (k = 5)	Worse: 526 (43.4)	Worse: 1,061 (35.9)	
	Better: 687 (56.6)	Better: 1,891 (64.1)	+
B) *Psychological capability*			
Memory (k = 3)	Worse: 1,148 (43.4)	Worse: 1,040 (32.0)	
	Better: 1,496 (56.6)	Better: 2,213 (68.0)	+
Health literacy (k = 3)	Worse: 470 (39.1)	Worse: 444 (27.1)	
	Better: 731 (60.9)	Better: 1,197 (72.9)	+
Opportunity:			
A) *Physical opportunity*			
Amount of green space (k = 3)	Lower: 5,582 (40.8)	Lower: 15,084 (52.8)	
	Higher: 8,086 (59.2)	Higher: 13,479 (47.2)	NS
Built physical activity facilities (k = 4)	Less: 3,706 (26.1)	Less: 13,165 (45.7)	
	More: 10,480 (73.9)	More: 15,647 (54.3)	+ ^*^
Natural physical activity facilities (k = 3)	Less: 9,872 (69.7)	Less: 24,213 (84.1)	
	More: 4,301 (30.3)	More: 4,565 (15.9)	NS
Walking and cycling infrastructure (k = 5)	Less: 3,725 (25.4)	Less: 18,148 (62.0)	
	More: 10,944 (74.6)	More: 11,132 (38.0)	+ ^*^
B) *Social opportunity*			
Dog ownership (k = 3)	No: 458 (66.8)	No: 608 (75.2)	
	Yes: 228 (33.2)	Yes: 200 (24.8)	NS
Social participation (k = 4)	Less: 3,335 (71.4)	Less: 1,657 (53.0)	
	More: 1,333 (28.6)	More: 1,467 (47.0)	+
Social support (k = 3)	Less: 125 (63.5)	Less: 96 (71.6)	
	More: 72 (36.5)	More: 38 (28.4)	NS
Health:			
A) *General health and wellbeing*			
Depressive symptoms, reversed (k = 10)	More: 7,589 (27.2)	More: 6,558 (46.8)	
	Less: 20,345 (72.8)	Less: 7,457 (53.2)	+
Perception of general health (k = 14)	Worse: 4,883 (42.1)	Worse: 3,979 (28.0)	
	Better: 6,714 (57.9)	Better: 10,254 (72.0)	+
Weight status, reversed (k = 19)	Higher BMI: 9,081 (62.9)	Higher BMI: 10,445 (62.6)	
	Lower BMI: 5,352 (37.1)	Lower BMI: 6,243 (37.4)	+
B) *Health behaviours*			
Alcohol consumption, reversed (k = 7)	More: 3,695 (48.1)	More: 3,962 (74.0)	
	Less: 3,982 (51.9)	Less: 1,392 (26.0)	–
Fruit and vegetable consumption (k = 3)	Less: 2,971 (64.4)	Less: 1,192 (63.6)	
	More: 1,644 (35.6)	More: 683 (36.4)	+
Sleep (k = 3)	Worse: 628 (52.5)	Worse: 610 (41.2)	
	Better: 568 (47.5)	Better: 870 (58.8)	NS
Smoking status, reversed (k = 9)	More smoking: 4,195 (52.6)	More smoking: 4,165 (44.3)	
	Less smoking: 3,778 (47.4)	Less smoking: 5,235 (55.7)	+ ^*^

*SES,* socio-economic status; *k,* number of studies; *n,* number of participants; *BMI,* body mass index; *NS,* not statistically significantly associated with physical activity

^*^Only statistically significant among lower-SES participants

## Discussion

The aim of this systematic review and meta-analysis was to synthesise evidence on modifiable correlates and determinants of physical activity in UK older adults, by SES. Of the exposures positively associated with physical activity, physical function, social participation, and perceived general health had the largest effect sizes. Associations were comparable among older adults of lower and higher SES in terms of direction and magnitude, apart from the presence of built physical activity facilities, access to walking and cycling infrastructure, and less smoking, which were positively associated with physical activity only in lower-SES participants. Moreover, a greater proportion of higher- compared to lower-SES participants were categorised in the “favourable” group (e.g., better versus worse perceived general health) for all three exposures under capability and six of the seven exposures under health, whereas the reverse was true for variables related to the physical opportunity sub-component of the COM-B model. Although this observation was based on frequency data (i.e., the raw number and proportion of participants exposed versus unexposed to each assessed correlate), an abundance of literature demonstrates that higher SES is associated with better health behaviours and outcomes across the lifespan [105, 106], which may act as important mechanisms underlying socio-economic inequalities in older adults’ physical activity levels.

Our results diverge from previous reviews on older adults across the socio-economic spectrum [14, 107], which concluded insufficient evidence for most intrapersonal or interpersonal correlates assessed in the present article, although pooled analyses were not conducted. Notably, we found that better physical and cognitive function, social participation, psychological wellbeing, and self-rated health were associated with more advantageous physical activity outcomes in both lower- and higher-SES sub-groups. Regarding the COM-B model, there was a lack of SES-disaggregated literature exploring the relationship between motivation and physical activity. As psychological constructs were frequently assessed in small-scale studies with restricted access, we relied on authors providing SES-stratified summary statistics for these to be eligible. Moreover, no associations were found for the physical opportunity variables, other than manmade facilities and walking and cycling infrastructure among lower-SES older adults, despite features of the built or natural environment purporting to support physical activity in older adults [15, 107, 108]. While it was not possible to formally test whether the magnitude of associations differed meaningfully by SES, the confidence intervals from the respective meta-analyses among lower versus higher-SES older adults overlapped. It is worth considering that older adults of higher SES are more likely to have access to a car to commute to facilities further away and may therefore be less dependent on local infrastructure to engage in leisure-time physical activity, which might explain the lack of associations in this sub-group [109]. Findings also suggest that healthy eating could induce corollary changes in physical activity, especially as lifestyle behaviours tend to cluster [110], although stronger research designs are required to infer causality. Given the multitude of seemingly pertinent modifiable variables, interventions for lower-SES older adults might benefit from a systems approach that emphasises the dynamic interconnections between factors driving physical activity behaviour [111].

To our knowledge, this systematic review with meta-analysis is the first to explore correlates of physical activity among older adults by SES and is strengthened by the number of included studies, facilitated through the extraction of data from openly available datasets, as well as supplementary analyses by original study authors. Nonetheless, there are some limitations to acknowledge. Firstly, the analyses did not account for indirect effects on physical activity. Some exposures could mediate associations between SES and physical activity [112], and/or between other exposures and physical activity, although the theoretical means to explain such associations are poorly understood. Furthermore, it was not possible to ascertain temporality, as most effect sizes came from cross-sectional designs, and some correlates, such as physical function, may act as both antecedents and consequences of physical activity behaviour. While dichotomising measures of SES increased comparability across studies for a given indicator (it should be noted that some indicators were necessarily binary) and enabled stratified meta-analyses to be conducted with sufficient participants in each sub-group, this approach provided limited information about underlying differences in the associations of correlates with physical activity behaviour among older adults located at each point or interval in the socio-economic continuum. It is also worth bearing in mind that some participants in the lower SES sub-group may have been categorised in the higher SES sub-group, and vice versa, if their assignment was based on a different socio-economic indicator or a different study’s cut-point for the same indicator. Moreover, exposure and outcome variables were often assessed using self-report instruments, with sparse evidence of validity or reliability, and effect sizes were based on bivariate rather than multivariate study-level data, which could have led to an overestimation of the coefficients. For example, the finding that alcohol consumption was positively associated with physical activity was likely confounded by participants’ levels of community engagement and/or their health status [113]. Given the observed heterogeneity in the collection of physical activity data, examining the correlates and determinants of lower-SES older adults’ participation across different intensities or modes of physical activity remains a future research priority. Finally, our review focuses on UK-based studies and findings may not be generalisable to other countries.

It is well-established that cross-national differences exist in cultural values and access to safe, affordable, and appropriate places in which to be physically active [16]. Research conducted among adults aged 50 years or over in low- and middle-income countries shows that associations of correlates spanning physical health, physical performance, mental health, health behaviours, and social cohesion with physical activity vary widely across countries [114]. While such studies may help to gauge the applicability of our findings to other settings, they do not typically discriminate between older adults of lower versus higher SES *within* countries, but rather focus only on differences in gross national income *between* countries. In addition, we argue that it may not be appropriate to generalise the results of this review to other countries in the absence of a standardised approach for operationalising SES. Common indicators of SES in the UK and other high-income countries might not be as contextually relevant in low- and middle-income countries, where other factors, including access to water and sanitation, nutrient deficiencies, and standard of living (e.g., asset ownership) often enter the equation [115]. It is also worth recognising that a household in the poorest wealth quintile in a higher-income country might be wealthier than a household in the richest wealth quintile in a lower-income country [115, 116].

Notwithstanding these considerations, there are several implications for practice, policy, and future research. First, while there were trivial differences in associations between the assessed correlates and physical activity among older adults of lower versus higher SES, disparities existed in the prevalence of these correlates, suggesting that programmes promoting social engagement, for instance, could be promising for lower-SES older adults, and may have knock-on effects on physical activity behaviour. This observation does not fully explain inequalities in physical activity, however, as exposures related to the physical opportunity sub-component of the COM-B model were more favourable among lower-SES participants (i.e., a greater proportion reported access to green space and physical activity facilities relative to higher-SES participants). Importantly, the correlates in this review are not exhaustive, as evidenced by the considerable number of independent variables that were assessed too infrequently to be meta-analysed. Many included studies used large cohort datasets, which were not explicitly designed to answer research questions on physical activity as an outcome. Closer inspection of study characteristics revealed that much research investigating associations between the environment and physical activity has operationalised exposures in terms of volume or access. This is noteworthy, as our concurrent systematic review of qualitative literature highlighted that lower-SES older adults perceived access and proximity to parks or walking routes as insufficient for inciting behaviour change, if crime, hazardous pedestrian infrastructure, and/or lack of beauty persisted [117]. The review reinforces the need for longitudinal, experimental, and mixed methods studies to examine whether these correlates are, in fact, causal determinants of physical activity and gauge their suitability as intervention targets. Furthermore, analyses were stratified based on various socio-economic indicators, but did not touch on intersectionality, such as the interaction between SES and other protected characteristics known to influence physical activity participation (e.g., gender, ethnicity, and disability); this remains an important area for future research.

## Conclusions

Overall, several correlates of physical activity in UK-based older adults were identified. Across the unique exposures in this review, eighteen were meta-analysed, showing that physical function, memory, health literacy, social participation, psychological wellbeing, perceptions of general health, lower body mass index, alcohol consumption, and fruit and vegetable consumption were positively associated with physical activity in older adults of lower and higher SES. Except for built physical activity facilities, walking and cycling infrastructure, and less smoking, which were positively associated with physical activity in participants of lower but not higher SES, there was little evidence that associations differed by SES. Rather, our results suggest it may be necessary to narrow discrepancies in the prevalence of the assessed correlates, or consider more nuanced concepts (e.g., perceptions of neighbourhood safety/aesthetics), to ensure that lower-SES older adults have equitable capability, opportunity, and motivation to participate in physical activity.

## Supplementary Information

Additional file 1. Search strategy for each database.

Additional file 2. Data extraction form.

Additional file 3. PRISMA guidelines.

Additional file 4. Mixed Methods Appraisal Tool (MMAT) guidance notes.

Additional file 5. Characteristics of the included studies.

Additional file 6. Risk of bias of the included studies contributing to each combined meta-analysis.

## Data Availability

The datasets generated and analysed during the current study, analytic code for the meta-analyses and extraction of additional data from publicly available datasets, results of sensitivity and sub-group analyses, funnel plots, and Egger’s regression tests, as well as a complete record of independent variables, higher-order exposures, and studies that were included in this review but not meta-analysed are available in the GitHub repository, https://github.com/OliviaMalkowski/Meta-analysis-PA-SES. To adhere to statistical disclosure standards, all data cells pertaining to a sample size of 30 or below have been censored. These data are however available from the authors upon reasonable request and with permission of the data owners, as outlined in the data availability statements of the original studies included in the systematic review.

## Abbreviations

CENTRAL: Cochrane Central Register of Controlled Trials
COM-B: Capability, Opportunity, Motivation, and Behaviour
GRADE: Grading of Recommendations Assessment, Development and Evaluation
ISRCTN: International Standard Randomised Controlled Trial Number
MMAT: Mixed Methods Appraisal Tool
PRISMA: Preferred Reporting Items for Systematic Reviews and Meta-Analyses
PROSPERO: International prospective register of systematic reviews
QAPAQ: Quality Assessment of Physical Activity Questionnaires
SES: Socio-economic status
UK: United Kingdom
