# Supplementary material for: Correlates and determinants of physical activity among older adults of lower versus higher socio-economic status: a systematic review and meta-analysis
Source: Int J Behav Nutr Phys Act. 2025 Jun 23;22:83. doi: 10.1186/s12966-025-01775-y (PMC12183859; doi:10.1186/s12966-025-01775-y)
Supplement: Supplementary file 1 — Additional file 1. Search strategy for each database. [file 12966_2025_1775_MOESM1_ESM.docx]

**Additional file 1.** Search strategy for each database

**Table S1.** MEDLINE search strategy

| **Block 1: Population** | |
| --- | --- |
| **1** | ***Population terms*** |
|  | "aged"[MeSH Terms] OR "old* age*"[Text Word] OR "aging"[MeSH Terms] OR "aging"[Text Word] OR "ageing"[Text Word] OR "middle aged"[MeSH Terms] OR "middle age*"[Text Word] OR "older adult*"[Text Word] OR "older person*"[Text Word] OR "older people*"[Text Word] OR "elder*"[Text Word] OR "senior"[Text Word] OR "seniors"[Text Word] OR "geriatric*"[Text Word] NOT ("child*"[Text Word] NOT "adult*"[Text Word]) NOT ("adolescen*" NOT "adult*") NOT ("animals"[MeSH Terms] NOT "humans"[MeSH Terms]) |
| **Block 2 : Outcome** | |
| **2** | ***Outcome terms*** |
|  | "motor activity"[MeSH Terms:noexp] OR "motor activit*"[Text Word] OR "exercise"[MeSH Terms] OR "exercis*"[Text Word] OR "sport*"[Text Word] OR "accelerometry"[MeSH Terms] OR "acceleromet*"[Text Word] OR "physical exertion"[MeSH Terms] OR "exertion*"[Text Word] OR "movement"[MeSH Terms:noexp] OR "movement*"[Text Word] OR "recreation*"[Text Word] OR "leisure activities"[MeSH Terms] OR "leisure activit*"[Text Word] OR "physical fitness"[MeSH Terms] OR "fitness"[Text Word] OR "walking"[MeSH Terms] OR "walk*"[Text Word] OR "commut*"[Text Word] OR "active transport*"[Text Word] OR "active travel*"[Text Word] OR "active living"[Text Word] OR "active lifestyle*"[Text Word] OR "cycle"[Text Word] OR "cycling"[Text Word] OR "bike*"[Text Word] OR "biking"[Text Word] OR "bicycl*"[Text Word] OR "swim*"[Text Word] OR "yoga"[MeSH Terms] OR "yoga"[Text Word] OR "pilates"[Text Word] OR "gardening"[Text Word] OR "running"[Text Word] OR "runner"[Text Word] OR "jogging"[Text Word] OR "danc*"[Text Word] OR "weight lift*"[Text Word] OR "physical train*"[Text Word] OR "exercise train*"[Text Word] OR "strength train*"[Text Word] OR "metabolic equivalent"[Text Word] OR "inactiv*"[Text Word] OR "physical activ*"[Text Word] OR "PAL"[Text Word] OR "workout"[Text Word] OR "MVPA"[Text Word] OR "IPAQ"[Text Word] OR "LTPA"[Text Word] OR "PAEE"[Text Word] OR "sedentary behavior"[MeSH Terms] OR "sedentar*"[Text Word] OR "screen time"[Text Word] OR "motor behavior*"[Text Word] OR "motor behaviour*"[Text Word] OR "steps"[Text Word] OR "stepping"[Text Word] OR "drama"[MeSH Terms] OR "drama"[Text Word] OR "theatre"[Text Word] OR "theater"[Text Word] OR "actor*"[Text Word] OR "actress*"[Text Word] OR "acting"[Text Word] OR "singing"[MeSH Terms] OR "sing"[Text Word] OR "singing"[Text Word] OR "artistic performance*"[Text Word] |
| **Block 3: Determinants/Correlates** | |
| **3** | ***Generic determinant/correlate terms*** |
|  | "determinant*"[Text Word] OR "correlat*"[Text Word] OR "influenc*"[Text Word] OR "associat*"[Text Word] OR "facilitat*"[Text Word] OR "barrier*"[Text Word] OR "mediat*"[Text Word] OR "moderat*"[Text Word] OR "contribut*"[Text Word] OR "modifier*"[Text Word] OR "confound*"[Text Word] OR "pattern*"[Text Word] OR "imped*"[Text Word] OR "predict*"[Text Word] OR "factor*"[Text Word] OR "characteristic*"[Text Word] OR "relationship*"[Text Word] OR "motive*"[Text Word] OR "motivator*"[Text Word] OR "psychosocial"[Text Word] |
| **4** | ***Policy terms*** |
|  | "public policy"[MeSH Terms] OR "public polic*"[Text Word] OR "health polic*"[Text Word] OR "road tax*"[Text Word] OR "toll road*"[Text Word] OR "standing break*"[Text Word] OR "workplace*"[Text Word] OR "sport program*"[Text Word] OR "stair design*"[Text Word] OR "physical activity counsel*"[Text Word] OR "workstation"[Text Word] OR "worksite"[Text Word] |
| **5** | ***Physical environment terms*** |
|  | "environment design"[MeSH Terms] OR "environment*"[Text Word] OR "coastal location*"[Text Word] OR "walkab*"[Text Word] OR "aesthetic*"[Text Word] OR "esthetic*"[Text Word] OR "safety"[Text Word] OR "equipment"[Text Word] OR "community design"[Text Word] OR "residence characteristics"[MeSH Terms] OR "residence characteristic*"[Text Word] OR "residential density"[Text Word] OR "neighborhood*"[Text Word] OR "neighbourhood*"[Text Word] OR "noise pollution"[Text Word] OR "urban*"[Text Word] OR "rural*"[Text Word] OR "community feature*"[Text Word] OR "community characteristic*"[Text Word] OR "weather"[MeSH Terms] OR "weather"[Text Word] OR "temperature*"[Text Word] OR "winter"[Text Word] OR "season*"[Text Word] OR "precipitation"[Text Word] OR "air pollution"[Text Word] OR "humid*"[Text Word] OR "public facilities"[MeSH Terms] OR "public facilit*"[Text Word] OR "recreation* facilit*"[Text Word] OR "recreation* location*"[Text Word] OR "recreation* space*"[Text Word] OR "sport* facilit*"[Text Word] OR "facilities"[Text Word] OR "leisure cent*"[Text Word] OR "litter"[Text Word] OR "pathway*"[Text Word] OR "footpath*"[Text Word] OR "sidewalk*"[Text Word] OR "public transport*"[Text Word] OR "public transit"[Text Word] OR "transport environment"[Text Word] OR "service*"[Text Word] OR "shop*"[Text Word] OR "streetlight*"[Text Word] OR "street light*"[Text Word] OR "traffic"[Text Word] OR "fitness centers"[MeSH Terms] OR "gym"[Text Word] OR "gyms"[Text Word] OR "fitness cent*"[Text Word] OR "recreation* cent*"[Text Word] OR "cycle friendly"[Text Word] OR "cycling friendly"[Text Word] OR "park*"[Text Word] OR "pavement*"[Text Word] OR "green space*"[Text Word] OR "blue space*"[Text Word] OR "greenery"[Text Word] OR "open space*"[Text Word] OR "walking trail*"[Text Word] OR "walking path*"[Text Word] OR "walking route*"[Text Word] OR "walking lane*"[Text Word] OR "walking infrastructure"[Text Word] OR "pedestrian trail*"[Text Word] OR "pedestrian path*"[Text Word] OR "pedestrian route*"[Text Word] OR "pedestrian lane*"[Text Word] OR "pedestrian infrastructure"[Text Word] OR "biking trail*"[Text Word] OR "biking path*"[Text Word] OR "biking route*"[Text Word] OR "biking lane*"[Text Word] OR "biking infrastructure"[Text Word] OR "bike trail*"[Text Word] OR "bike path*"[Text Word] OR "bike route*"[Text Word] OR "bike lane*"[Text Word] OR "bike infrastructure"[Text Word] OR "bicycle trail*"[Text Word] OR "bicycle path*"[Text Word] OR "bicycle route*"[Text Word] OR "bicycle lane*"[Text Word] OR "bicycle infrastructure"[Text Word] OR "cycling trail*"[Text Word] OR "cycling path*"[Text Word] OR "cycling route*"[Text Word] OR "cycling lane*"[Text Word] OR "cycling infrastructure"[Text Word] OR "cycle trail*"[Text Word] OR "cycle path*"[Text Word] OR "cycle route*"[Text Word] OR "cycle lane*"[Text Word] OR "cycle infrastructure"[Text Word] OR "road connectivity"[Text Word] OR "street connectivity"[Text Word] OR "land use mix*"[Text Word] OR "distance*"[Text Word] OR "destination*"[Text Word] OR "vandalism"[Text Word] OR "exercis* facilit*"[Text Word] |
| **6** | ***Interpersonal terms*** |
|  | "social environment"[MeSH Terms] OR "social environment*"[Text Word] OR "social connect*"[Text Word] OR "social trust"[Text Word] OR "social cohesion"[Text Word] OR "social participation"[Text Word] OR "social function*"[Text Word] OR "social tie*"[Text Word] OR "community network*"[Text Word] OR "social network*"[Text Word] OR "crime"[MeSH Terms] OR "crime*"[Text Word] OR "criminal*"[Text Word] OR "violen*"[Text Word] OR "social support"[Text Word] OR "family support"[Text Word] OR "peer support"[Text Word] OR "dog owner*"[Text Word] OR "perceived peer attitude*"[Text Word] OR "role model*"[Text Word] OR "parent* physical activ*"[Text Word] OR "parent* activit*"[Text Word] OR "sibling* physical activ*"[Text Word] OR "sibling* activit*"[Text Word] OR "social isolation"[MeSH Terms] OR "social isolat*"[Text Word] OR "loneliness"[Text Word] OR "culture"[MeSH Terms] OR "social norm*"[Text Word] OR "cultur*"[Text Word] OR "social capital"[Text Word] OR "social programming"[Text Word] OR "communication"[MeSH Terms] OR "communicat*"[Text Word] |
| **7** | ***Intrapersonal terms*** |
|  | "personality"[MeSH Terms] OR "self-efficac*"[Text Word] OR "perceived competenc*"[Text Word] OR "intention*"[Text Word] OR "motivation"[MeSH Terms] OR "motivation*"[Text Word] OR "enjoyment"[Text Word] OR "planning"[Text Word] OR "action plan*"[Text Word] OR "goal-setting"[Text Word] OR "self-regulat*"[Text Word] OR "habit strength"[Text Word] OR "outcome expecta*"[Text Word] OR "outcome reali*"[Text Word] OR "perception*"[Text Word] OR "preference*"[Text Word] OR "health status"[MeSH Terms] OR "health status"[Text Word] OR "physical health"[Text Word] OR "mental health"[Text Word] OR "wellbeing"[Text Word] OR "well-being"[Text Word] OR "mobilit*"[Text Word] OR "stiff*"[Text Word] OR "physical performance"[Text Word] OR "physical function"[Text Word] OR "attitude to health"[MeSH Terms] OR "knowledge"[Text Word] OR "belief*"[Text Word] OR "attitude*"[Text Word] OR "health behavior"[MeSH Terms] OR "health behavior*"[Text Word] OR "health behaviour*"[Text Word] OR "personalit*"[Text Word] OR "impulsive*"[Text Word] OR "cognitive abilit*"[Text Word] OR "cognition*"[Text Word] OR "temperament*"[Text Word] OR "perceived behavioral control"[Text Word] OR "perceived behavioural control"[Text Word] OR "perceived physical condition"[Text Word] OR "locus of control"[Text Word] OR "self-esteem"[Text Word] OR "self-concept"[Text Word] OR "self-worth"[Text Word] OR "self-accept*"[Text Word] OR "satisfaction"[Text Word] OR "neurotic*"[Text Word] OR "smok*"[Text Word] OR "alcohol*"[Text Word] OR "food intake"[Text Word] OR "nutritional intake"[Text Word] OR "energy intake"[Text Word] OR "nutrient intake"[Text Word] OR "diet*"[Text Word] OR "stress*"[Text Word] OR "anxiet*"[Text Word] OR "anxious*"[Text Word] OR "depress*"[Text Word] OR "mood*"[Text Word] OR "sleep*"[Text Word] OR "vitality"[Text Word] OR "quality-of-life"[Text Word] OR "pain*"[Text Word] OR "fatigue*"[Text Word] OR "body mass index"[Text Word] OR "BMI"[Text Word] OR "weight status"[Text Word] OR "overweight"[Text Word] OR "underweight"[Text Word] OR "obes*"[Text Word] OR "internet access"[Text Word] OR "internet use"[Text Word] OR "internet usage"[Text Word] OR "health literacy"[Text Word] |
| **Block 4: United Kingdom (UK)** | |
| **8** | ***UK terms*** |
|  | "uk"[Text Word] OR "u.k."[Text Word] OR "united kingdom"[Text Word] OR "england*"[Text Word] OR "english*"[Text Word] OR "scotland*"[Text Word] OR "scottish*"[Text Word] OR "wales"[Text Word] OR "south wales"[Text Word] OR "welsh*"[Text Word] OR "northern ireland*"[Text Word] OR "northern irish*"[Text Word] OR "gb"[Text Word] OR "g.b."[Text Word] OR "britain*"[Text Word] OR "british*"[Text Word] NOT "new england"[Text Word] NOT "new south wales"[Text Word] NOT "british columbia"[Text Word] |
| **9** | ***Cross-national terms*** |
|  | "crosscultural*"[Title] OR "cross-cultural*"[Title] OR "crossnational*"[Title] OR "cross-national*"[Title] OR "crossborder*"[Title] OR "cross-border*"[Title] OR "multinational*"[Title] OR "multi-national*"[Title] OR "multicultural*"[Title] OR "multi-cultural*"[Title] OR "international*"[Title] OR "transnational*"[Title] OR "trans-national*"[Title] OR "multicountry"[Title] OR "multi-country"[Title] OR "multiple countr*"[Title] OR "intercountry"[Title] OR "inter-country"[Title] OR "country comparison*"[Title] OR "countries"[Title] OR "continent*"[Title] OR "intercontinent*"[Title] OR "world*"[Title] OR "global*"[Title] OR "Europe*"[Title] OR "Western*"[Title] OR "Commonwealth"[Title] OR "cohorts"[Title] OR "studies"[Title] OR "harmoni*"[Title] |
| **Block 5: Filters** | |
| **10** | ***Filters*** |
|  | "medline"[Filter] AND "english"[Language] NOT "review"[Publication Type] NOT "systematic review"[Publication Type] NOT "meta-analysis"[Publication Type] NOT "editorial"[Publication Type] |
| **Block 6: Socio-economic status** | |
| **11** | ***Socio-economic status terms*** |
|  | "occupation*"[Text Word] OR "income"[Text Word] OR "educat*"[Text Word] OR "socioeconomic factors"[MeSH Terms] OR "socioeconomic*"[Text Word] OR "socio-economic*"[Text Word] OR "social class*"[Text Word] OR "ses"[Text Word] OR "disadvantag*"[Text Word] OR "underserved"[Text Word] OR "privilege*"[Text Word] OR "depriv*"[Text Word] OR "poverty"[Text Word] OR "inequalit*"[Text Word] OR "low socio*"[Text Word] OR "high socio*"[Text Word] OR "poverty area*"[Text Word] OR "IMD"[Text Word] OR "dispar*"[Text Word] OR "employ*"[Text Word] |
| **Proximity operators: Combining searches** | |
| **12** | (3 OR 4 OR 5 OR 6 OR 7) |
| **13** | (8 OR 9) |
| **14** | 1 AND 2 AND 10 AND 11 AND 12 AND 13 |

*Captures alternative word endings

*MeSH*, Medical Subject Headings; *noexp*, no explosion.

**Table S2.** Embase search strategy

| **Block 1: Population** | |
| --- | --- |
| **1** | ***Population terms*** |
|  | ('old* age*':ti,ab,kw OR 'aging':ti,ab,kw OR 'ageing':ti,ab,kw OR 'middle age*':ti,ab,kw OR 'older adult*':ti,ab,kw OR 'older person*':ti,ab,kw OR 'older people*':ti,ab,kw OR 'elder*':ti,ab,kw OR 'senior':ti,ab,kw OR 'seniors':ti,ab,kw OR 'geriatric*':ti,ab,kw) NOT ('child*':ti,ab,kw NOT 'adult*':ti,ab,kw) NOT ('adolescen*':ti,ab,kw NOT 'adult*':ti,ab,kw) OR 'aged'/exp OR 'aging'/exp OR 'middle aged'/exp |
| **Block 2 : Outcome** | |
| **2** | ***Outcome terms*** |
|  | 'motor activit*':ti,ab,kw OR 'exercis*':ti,ab,kw OR 'sport*':ti,ab,kw OR 'acceleromet*':ti,ab,kw OR 'exertion*':ti,ab,kw OR 'movement*':ti,ab,kw OR 'recreation*':ti,ab,kw OR 'leisure activit*':ti,ab,kw OR 'fitness':ti,ab,kw OR 'walk*':ti,ab,kw OR 'commut*':ti,ab,kw OR 'active transport*':ti,ab,kw OR 'active travel*':ti,ab,kw OR 'active living':ti,ab,kw OR 'active lifestyle*':ti,ab,kw OR 'cycle':ti,ab,kw OR 'cycling':ti,ab,kw OR 'bike*':ti,ab,kw OR 'biking':ti,ab,kw OR 'bicycl*':ti,ab,kw OR 'swim*':ti,ab,kw OR 'yoga':ti,ab,kw OR 'pilates':ti,ab,kw OR 'gardening':ti,ab,kw OR 'running':ti,ab,kw OR 'runner':ti,ab,kw OR 'jogging':ti,ab,kw OR 'danc*':ti,ab,kw OR 'weight lift*':ti,ab,kw OR 'physical train*':ti,ab,kw OR 'exercise train*':ti,ab,kw OR 'strength train*':ti,ab,kw OR 'metabolic equivalent':ti,ab,kw OR 'inactiv*':ti,ab,kw OR 'physical activ*':ti,ab,kw OR 'pal':ti,ab,kw OR 'workout':ti,ab,kw OR 'mvpa':ti,ab,kw OR 'ipaq':ti,ab,kw OR 'ltpa':ti,ab,kw OR 'paee':ti,ab,kw OR 'sedentar*':ti,ab,kw OR 'screen time':ti,ab,kw OR 'motor behavior*':ti,ab,kw OR 'motor behaviour*':ti,ab,kw OR 'steps':ti,ab,kw OR 'stepping':ti,ab,kw OR 'drama':ti,ab,kw OR 'theatre':ti,ab,kw OR 'theater':ti,ab,kw OR 'actor*':ti,ab,kw OR 'actress*':ti,ab,kw OR 'acting':ti,ab,kw OR 'sing':ti,ab,kw OR 'singing':ti,ab,kw OR 'artistic performance*':ti,ab,kw OR 'motor activity'/de OR 'exercise'/exp OR 'accelerometry'/exp OR ('movement'/de AND 'physiology'/de) OR 'leisure'/exp OR 'fitness'/exp OR 'walking'/exp OR 'sedentary lifestyle'/exp OR 'physical activity'/exp OR 'literature'/exp OR 'singing'/exp OR 'yoga'/exp |
| **Block 3: Determinants/Correlates** | |
| **3** | ***Generic determinant/correlate terms*** |
|  | 'determinant*':ti,ab,kw OR 'correlat*':ti,ab,kw OR 'influenc*':ti,ab,kw OR 'associat*':ti,ab,kw OR 'facilitat*':ti,ab,kw OR 'barrier*':ti,ab,kw OR 'mediat*':ti,ab,kw OR 'moderat*':ti,ab,kw OR 'contribut*':ti,ab,kw OR 'modifier*':ti,ab,kw OR 'confound*':ti,ab,kw OR 'pattern*':ti,ab,kw OR 'imped*':ti,ab,kw OR 'predict*':ti,ab,kw OR 'factor*':ti,ab,kw OR 'characteristic*':ti,ab,kw OR 'relationship*':ti,ab,kw OR 'motive*':ti,ab,kw OR 'motivator*':ti,ab,kw OR 'psychosocial':ti,ab,kw |
| **4** | ***Policy terms*** |
|  | 'public polic*':ti,ab,kw OR 'health polic*':ti,ab,kw OR 'road tax*':ti,ab,kw OR 'toll road*':ti,ab,kw OR 'standing break*':ti,ab,kw OR 'workplace*':ti,ab,kw OR 'sport program*':ti,ab,kw OR 'stair design*':ti,ab,kw OR 'physical activity counsel*':ti,ab,kw OR 'workstation':ti,ab,kw OR 'worksite':ti,ab,kw OR 'public policy'/exp |
| **5** | ***Physical environment terms*** |
|  | 'environment*':ti,ab,kw OR 'coastal location*':ti,ab,kw OR 'walkab*':ti,ab,kw OR 'aesthetic*':ti,ab,kw OR 'esthetic*':ti,ab,kw OR 'safety':ti,ab,kw OR 'equipment':ti,ab,kw OR 'community design':ti,ab,kw OR 'residence characteristic*':ti,ab,kw OR 'residential density':ti,ab,kw OR 'neighborhood*':ti,ab,kw OR 'neighbourhood*':ti,ab,kw OR 'noise pollution':ti,ab,kw OR 'urban*':ti,ab,kw OR 'rural*':ti,ab,kw OR 'community feature*':ti,ab,kw OR 'community characteristic*':ti,ab,kw OR 'weather':ti,ab,kw OR 'temperature*':ti,ab,kw OR 'winter':ti,ab,kw OR 'season*':ti,ab,kw OR 'precipitation':ti,ab,kw OR 'air pollution':ti,ab,kw OR 'humid*':ti,ab,kw OR 'public facilit*':ti,ab,kw OR 'recreation* facilit*':ti,ab,kw OR 'recreation* location*':ti,ab,kw OR 'recreation* space*':ti,ab,kw OR 'sport* facilit*':ti,ab,kw OR 'facilities':ti,ab,kw OR 'leisure cent*':ti,ab,kw OR 'litter':ti,ab,kw OR 'pathway*':ti,ab,kw OR 'footpath*':ti,ab,kw OR 'sidewalk*':ti,ab,kw OR 'public transport*':ti,ab,kw OR 'public transit':ti,ab,kw OR 'transport environment':ti,ab,kw OR 'service*':ti,ab,kw OR 'shop*':ti,ab,kw OR 'streetlight*':ti,ab,kw OR 'street light*':ti,ab,kw OR 'traffic':ti,ab,kw OR 'gym':ti,ab,kw OR 'gyms':ti,ab,kw OR 'fitness cent*':ti,ab,kw OR 'recreation* cent*':ti,ab,kw OR 'cycle friendly':ti,ab,kw OR 'cycling friendly':ti,ab,kw OR 'park*':ti,ab,kw OR 'pavement*':ti,ab,kw OR 'green space*':ti,ab,kw OR 'blue space*':ti,ab,kw OR 'greenery':ti,ab,kw OR 'open space*':ti,ab,kw OR 'walking trail*':ti,ab,kw OR 'walking path*':ti,ab,kw OR 'walking route*':ti,ab,kw OR 'walking lane*':ti,ab,kw OR 'walking infrastructure':ti,ab,kw OR 'pedestrian trail*':ti,ab,kw OR 'pedestrian path*':ti,ab,kw OR 'pedestrian route*':ti,ab,kw OR 'pedestrian lane*':ti,ab,kw OR 'pedestrian infrastructure':ti,ab,kw OR 'biking trail*':ti,ab,kw OR 'biking path*':ti,ab,kw OR 'biking route*':ti,ab,kw OR 'biking lane*':ti,ab,kw OR 'biking infrastructure':ti,ab,kw OR 'bike trail*':ti,ab,kw OR 'bike path*':ti,ab,kw OR 'bike route*':ti,ab,kw OR 'bike lane*':ti,ab,kw OR 'bike infrastructure':ti,ab,kw OR 'bicycle trail*':ti,ab,kw OR 'bicycle path*':ti,ab,kw OR 'bicycle route*':ti,ab,kw OR 'bicycle lane*':ti,ab,kw OR 'bicycle infrastructure':ti,ab,kw OR 'cycling trail*':ti,ab,kw OR 'cycling path*':ti,ab,kw OR 'cycling route*':ti,ab,kw OR 'cycling lane*':ti,ab,kw OR 'cycling infrastructure':ti,ab,kw OR 'cycle trail*':ti,ab,kw OR 'cycle path*':ti,ab,kw OR 'cycle route*':ti,ab,kw OR 'cycle lane*':ti,ab,kw OR 'cycle infrastructure':ti,ab,kw OR 'road connectivity':ti,ab,kw OR 'street connectivity':ti,ab,kw OR 'land use mix*':ti,ab,kw OR 'distance*':ti,ab,kw OR 'destination*':ti,ab,kw OR 'vandalism':ti,ab,kw OR 'exercis* facilit*':ti,ab,kw OR 'environmental planning'/exp OR 'demography'/exp OR 'weather'/exp OR 'fitness center'/exp |
| **6** | ***Interpersonal terms*** |
|  | 'social environment*':ti,ab,kw OR 'social connect*':ti,ab,kw OR 'social trust':ti,ab,kw OR 'social cohesion':ti,ab,kw OR 'social participation':ti,ab,kw OR 'social function*':ti,ab,kw OR 'social tie*':ti,ab,kw OR 'community network*':ti,ab,kw OR 'social network*':ti,ab,kw OR 'crime*':ti,ab,kw OR 'criminal*':ti,ab,kw OR 'violen*':ti,ab,kw OR 'social support':ti,ab,kw OR 'family support':ti,ab,kw OR 'peer support':ti,ab,kw OR 'dog owner*':ti,ab,kw OR 'perceived peer attitude*':ti,ab,kw OR 'role model*':ti,ab,kw OR 'parent* physical activ*':ti,ab,kw OR 'parent* activit*':ti,ab,kw OR 'sibling* physical activ*':ti,ab,kw OR 'sibling* activit*':ti,ab,kw OR 'social isolat*':ti,ab,kw OR 'loneliness':ti,ab,kw OR 'social norm*':ti,ab,kw OR 'cultur*':ti,ab,kw OR 'social capital':ti,ab,kw OR 'social programming':ti,ab,kw OR 'communicat*':ti,ab,kw OR 'social environment'/exp OR 'crime'/exp OR 'social isolation'/exp OR 'cultural anthropology'/exp OR 'interpersonal communication'/exp |
| **7** | ***Intrapersonal terms*** |
|  | 'self-efficac*':ti,ab,kw OR 'perceived competenc*':ti,ab,kw OR 'intention*':ti,ab,kw OR 'motivation*':ti,ab,kw OR 'enjoyment':ti,ab,kw OR 'planning':ti,ab,kw OR 'action plan*':ti,ab,kw OR 'goal-setting':ti,ab,kw OR 'self-regulat*':ti,ab,kw OR 'habit strength':ti,ab,kw OR 'outcome expecta*':ti,ab,kw OR 'outcome reali*':ti,ab,kw OR 'perception*':ti,ab,kw OR 'preference*':ti,ab,kw OR 'health status':ti,ab,kw OR 'physical health':ti,ab,kw OR 'mental health':ti,ab,kw OR 'wellbeing':ti,ab,kw OR 'well-being':ti,ab,kw OR 'mobilit*':ti,ab,kw OR 'stiff*':ti,ab,kw OR 'physical performance':ti,ab,kw OR 'physical function':ti,ab,kw OR 'knowledge':ti,ab,kw OR 'belief*':ti,ab,kw OR 'attitude*':ti,ab,kw OR 'health behavior*':ti,ab,kw OR 'health behaviour*':ti,ab,kw OR 'personalit*':ti,ab,kw OR 'impulsive*':ti,ab,kw OR 'cognitive abilit*':ti,ab,kw OR 'cognition*':ti,ab,kw OR 'temperament*':ti,ab,kw OR 'perceived behavioral control':ti,ab,kw OR 'perceived behavioural control':ti,ab,kw OR 'perceived physical condition':ti,ab,kw OR 'locus of control':ti,ab,kw OR 'self-esteem':ti,ab,kw OR 'self-concept':ti,ab,kw OR 'self-worth':ti,ab,kw OR 'self-accept*':ti,ab,kw OR 'satisfaction':ti,ab,kw OR 'neurotic*':ti,ab,kw OR 'smok*':ti,ab,kw OR 'alcohol*':ti,ab,kw OR 'food intake':ti,ab,kw OR 'nutritional intake':ti,ab,kw OR 'energy intake':ti,ab,kw OR 'nutrient intake':ti,ab,kw OR 'diet*':ti,ab,kw OR 'stress*':ti,ab,kw OR 'anxiet*':ti,ab,kw OR 'anxious*':ti,ab,kw OR 'depress*':ti,ab,kw OR 'mood*':ti,ab,kw OR 'sleep*':ti,ab,kw OR 'vitality':ti,ab,kw OR 'quality-of-life':ti,ab,kw OR 'pain*':ti,ab,kw OR 'fatigue*':ti,ab,kw OR 'body mass index':ti,ab,kw OR 'bmi':ti,ab,kw OR 'weight status':ti,ab,kw OR 'overweight':ti,ab,kw OR 'underweight':ti,ab,kw OR 'obes*':ti,ab,kw OR 'internet access':ti,ab,kw OR 'internet use':ti,ab,kw OR 'internet usage':ti,ab,kw OR 'health literacy':ti,ab,kw OR 'personality'/exp OR 'motivation'/exp OR 'health status'/exp OR 'attitude to health'/exp OR 'health behavior'/exp |
| **Block 4: United Kingdom (UK)** | |
| **8** | ***UK terms*** |
|  | ('uk':ti,ab,kw OR 'u.k.':ti,ab,kw OR 'united kingdom':ti,ab,kw OR 'england*':ti,ab,kw OR 'english*':ti,ab,kw OR 'scotland*':ti,ab,kw OR 'scottish*':ti,ab,kw OR 'wales':ti,ab,kw OR 'south wales':ti,ab,kw OR 'welsh*':ti,ab,kw OR 'northern ireland*':ti,ab,kw OR 'northern irish*':ti,ab,kw OR 'gb':ti,ab,kw OR 'g.b.':ti,ab,kw OR 'britain*':ti,ab,kw OR 'british*':ti,ab,kw) NOT 'new england':ti,ab,kw NOT 'new south wales':ti,ab,kw NOT 'british columbia':ti,ab,kw |
| **9** | ***Cross-national terms*** |
|  | 'crosscultural*':ti OR 'cross-cultural*':ti OR 'crossnational*':ti OR 'cross-national*':ti OR 'crossborder*':ti OR 'cross-border*':ti OR 'multinational*':ti OR 'multi-national*':ti OR 'multicultural*':ti OR 'multi-cultural*':ti OR 'international*':ti OR 'transnational*':ti OR 'trans-national*':ti OR 'multicountry':ti OR 'multi-country':ti OR 'multiple countr*':ti OR 'intercountry':ti OR 'inter-country':ti OR 'country comparison*':ti OR 'countries':ti OR 'continent*':ti OR 'intercontinent*':ti OR 'world*':ti OR 'global*':ti OR 'europe*':ti OR 'western*':ti OR 'commonwealth':ti OR 'cohorts':ti OR 'studies':ti OR 'harmoni*':ti |
| **Block 5: Filters** | |
| **10** | ***Filters*** |
|  | ([embase]/lim OR [embase classic]/lim) AND [english]/lim NOT 'conference abstract'/it NOT editorial/it NOT review/it |
| **Block 6: Socio-economic status** | |
| **11** | ***Socio-economic status terms*** |
|  | 'occupation*':ti,ab,kw OR 'income':ti,ab,kw OR 'educat*':ti,ab,kw OR 'socioeconomic*':ti,ab,kw OR 'socio-economic*':ti,ab,kw OR 'social class*':ti,ab,kw OR 'ses':ti,ab,kw OR 'disadvantag*':ti,ab,kw OR 'underserved':ti,ab,kw OR 'privilege*':ti,ab,kw OR 'depriv*':ti,ab,kw OR 'poverty':ti,ab,kw OR 'inequalit*':ti,ab,kw OR 'low socio*':ti,ab,kw OR 'high socio*':ti,ab,kw OR 'poverty area*':ti,ab,kw OR 'imd':ti,ab,kw OR 'dispar*':ti,ab,kw OR 'employ*':ti,ab,kw OR 'socioeconomics'/exp |
| **Proximity operators: Combining searches** | |
| **12** | (3 OR 4 OR 5 OR 6 OR 7) |
| **13** | (8 OR 9) |
| **14** | 1 AND 2 AND 10 AND 11 AND 12 AND 13 |

*Captures alternative word endings

*de*, no explosion; *exp*, exploded term.

**Table S3.** Web of Science search strategy

| **Block 1: Population** | |
| --- | --- |
| **1** | ***Population terms*** |
|  | "old* age*" OR "aging" OR "ageing" OR "middle age*" OR "older adult*" OR "older person*" OR "older people*" OR "elder*" OR "senior" OR "seniors" OR "geriatric*" NOT ("child*" NOT "adult*") NOT ("adolescen*" NOT "adult*") |
| **Block 2 : Outcome** | |
| **2** | ***Outcome terms*** |
|  | "motor activit*" OR "exercis*" OR "sport*" OR "acceleromet*" OR "exertion*" OR "movement*" OR "recreation*" OR "leisure activit*" OR "fitness" OR "walk*" OR "commut*" OR "active transport*" OR "active travel*" OR "active living" OR "active lifestyle*" OR "cycle" OR "cycling" OR "bike*" OR "biking" OR "bicycl*" OR "swim*" OR "yoga" OR "pilates" OR "gardening" OR "running" OR "runner" OR "jogging" OR "danc*" OR "weight lift*" OR "physical train*" OR "exercise train*" OR "strength train*" OR "metabolic equivalent" OR "inactiv*" OR "physical activ*" OR "PAL" OR "workout" OR "MVPA" OR "IPAQ" OR "LTPA" OR "PAEE" OR "sedentar*" OR "screen time" OR "motor behavior*" OR "motor behaviour*" OR "steps" OR "stepping" OR "drama" OR "theatre" OR "theater" OR "actor*" OR "actress*" OR "acting" OR "sing" OR "singing" OR "artistic performance*" |
| **Block 3: Determinants/Correlates** | |
| **3** | ***Generic determinant/correlate terms*** |
|  | "determinant*" OR "correlat*" OR "influenc*" OR "associat*" OR "facilitat*" OR "barrier*" OR "mediat*" OR "moderat*" OR "contribut*" OR "modifier*" OR "confound*" OR "pattern*" OR "imped*" OR "predict*" OR "factor*" OR "characteristic*" OR "relationship*" OR "motive*" OR "motivator*" OR "psychosocial" |
| **4** | ***Policy terms*** |
|  | "public polic*" OR "health polic*" OR "road tax*" OR "toll road*" OR "standing break*" OR "workplace*" OR "sport program*" OR "stair design*" OR "physical activity counsel*" OR "workstation" OR "worksite" |
| **5** | ***Physical environment terms*** |
|  | "environment*" OR "coastal location*" OR "walkab*" OR "aesthetic*" OR "esthetic*" OR "safety" OR "equipment" OR "community design" OR "residence characteristic*" OR "residential density" OR "neighborhood*" OR "neighbourhood*" OR "noise pollution" OR "urban*" OR "rural*" OR "community feature*" OR "community characteristic*" OR "weather" OR "temperature*" OR "winter" OR "season*" OR "precipitation" OR "air pollution" OR "humid*" OR "public facilit*" OR "recreation* facilit*" OR "recreation* location*" OR "recreation* space*" OR "sport* facilit*" OR "facilities" OR "leisure cent*" OR "litter" OR "pathway*" OR "footpath*" OR "sidewalk*" OR "public transport*" OR "public transit" OR "transport environment" OR "service*" OR "shop*" OR "streetlight*" OR "street light*" OR "traffic" OR "gym" OR "gyms" OR "fitness cent*" OR "recreation* cent*" OR "cycle friendly" OR "cycling friendly" OR "park*" OR "pavement*" OR "green space*" OR "blue space*" OR "greenery" OR "open space*" OR "walking trail*" OR "walking path*" OR "walking route*" OR "walking lane*" OR "walking infrastructure" OR "pedestrian trail*" OR "pedestrian path*" OR "pedestrian route*" OR "pedestrian lane*" OR "pedestrian infrastructure" OR "biking trail*" OR "biking path*" OR "biking route*" OR "biking lane*" OR "biking infrastructure" OR "bike trail*" OR "bike path*" OR "bike route*" OR "bike lane*" OR "bike infrastructure" OR "bicycle trail*" OR "bicycle path*" OR "bicycle route*" OR "bicycle lane*" OR "bicycle infrastructure" OR "cycling trail*" OR "cycling path*" OR "cycling route*" OR "cycling lane*" OR "cycling infrastructure" OR "cycle trail*" OR "cycle path*" OR "cycle route*" OR "cycle lane*" OR "cycle infrastructure" OR "road connectivity" OR "street connectivity" OR "land use mix*" OR "distance*" OR "destination*" OR "vandalism" OR "exercis* facilit*" |
| **6** | ***Interpersonal terms*** |
|  | "social environment*" OR "social connect*" OR "social trust" OR "social cohesion" OR "social participation" OR "social function*" OR "social tie*" OR "community network*" OR "social network*" OR "crime*" OR "criminal*" OR "violen*" OR "social support" OR "family support" OR "peer support" OR "dog owner*" OR "perceived peer attitude*" OR "role model*" OR "parent* physical activ*" OR "parent* activit*" OR "sibling* physical activ*" OR "sibling* activit*" OR "social isolat*" OR "loneliness" OR "social norm*" OR "cultur*" OR "social capital" OR "social programming" OR "communicat*" |
| **7** | ***Intrapersonal terms*** |
|  | "self-efficac*" OR "perceived competenc*" OR "intention*" OR "motivation*" OR "enjoyment" OR "planning" OR "action plan*" OR "goal-setting" OR "self-regulat*" OR "habit strength" OR "outcome expecta*" OR "outcome reali*" OR "perception*" OR "preference*" OR "health status" OR "physical health" OR "mental health" OR "wellbeing" OR "well-being" OR "mobilit*" OR "stiff*" OR "physical performance" OR "physical function" OR "knowledge" OR "belief*" OR "attitude*" OR "health behavior*" OR "health behaviour*" OR "personalit*" OR "impulsive*" OR "cognitive abilit*" OR "cognition*" OR "temperament*" OR "perceived behavioral control" OR "perceived behavioural control" OR "perceived physical condition" OR "locus of control" OR "self-esteem" OR "self-concept" OR "self-worth" OR "self-accept*" OR "satisfaction" OR "neurotic*" OR "smok*" OR "alcohol*" OR "food intake" OR "nutritional intake" OR "energy intake" OR "nutrient intake" OR "diet*" OR "stress*" OR "anxiet*" OR "anxious*" OR "depress*" OR "mood*" OR "sleep*" OR "vitality" OR "quality-of-life" OR "pain*" OR "fatigue*" OR "body mass index" OR "BMI" OR "weight status" OR "overweight" OR "underweight" OR "obes*" OR "internet access" OR "internet use" OR "internet usage" OR "health literacy" |
| **Block 4: United Kingdom (UK)** | |
| **8** | ***UK terms*** |
|  | "uk" OR "u.k." OR "united kingdom" OR "england*" OR "english*" OR "scotland*" OR "scottish*" OR "wales" OR "south wales" OR "welsh*" OR "northern ireland*" OR "northern irish*" OR "gb" OR "g.b." OR "britain*" OR "british*" NOT "new england" NOT "new south wales" NOT "british columbia" |
| **9** | ***Cross-national terms*** |
|  | "crosscultural*" OR "cross-cultural*" OR "crossnational*" OR "cross-national*" OR "crossborder*" OR "cross-border*" OR "multinational*" OR "multi-national*" OR "multicultural*" OR "multi-cultural*" OR "international*" OR "transnational*" OR "trans-national*" OR "multicountry" OR "multi-country" OR "multiple countr*" OR "intercountry" OR "inter-country" OR "country comparison*" OR "countries" OR "continent*" OR "intercontinent*" OR "world*" OR "global*" OR "Europe*" OR "Western*" OR "Commonwealth" OR "cohorts" OR "studies" OR "harmoni*" |
| **Block 5: Filters** | |
| **10** | ***Filters*** |
|  | Book Chapters or Review Articles (Exclude – Document Types) and English (Languages) |
| **Block 6: Socio-economic status** | |
| **11** | ***Socio-economic status terms*** |
|  | "occupation*" OR "income" OR "educat*" OR "socioeconomic*" OR "socio-economic*" OR "social class*" OR "ses" OR "disadvantag*" OR "underserved" OR "privilege*" OR "depriv*" OR "poverty" OR "inequalit*" OR "low socio*" OR "high socio*" OR "poverty area*" OR "IMD" OR "dispar*" OR "employ*" |
| **Proximity operators: Combining searches** | |
| **12** | (3 OR 4 OR 5 OR 6 OR 7) |
| **13** | (8 OR 9) |
| **14** | 1 AND 2 AND 10 AND 11 AND 12 AND 13 |

*Captures alternative word endings

**Table S4.** Cochrane Central Register of Controlled Trials (CENTRAL) search strategy (in Trials only)

| **Block 1: Population** | |
| --- | --- |
|  | ***Population terms*** |
| **#1** | MeSH descriptor: [Aged] explode all trees |
| **#2** | MeSH descriptor: [Aging] explode all trees |
| **#3** | MeSH descriptor: [Middle Aged] explode all trees |
| **#4** | ("old* age*" OR "aging" OR "ageing" OR "middle age*" OR "older adult*" OR "older person*" OR "older people*" OR "elder*" OR "senior" OR "seniors" OR "geriatric*" NOT ("child*" NOT "adult*") NOT ("adolescen*" NOT "adult*")):ti,ab,kw |
| **#5** | {OR #1-#4} |
| **Block 2 : Outcome** | |
|  | ***Outcome terms*** |
| **#6** | MeSH descriptor: [Motor Activity] this term only |
| **#7** | MeSH descriptor: [Exercise] explode all trees |
| **#8** | MeSH descriptor: [Accelerometry] explode all trees |
| **#9** | MeSH descriptor: [Physical Exertion] explode all trees |
| **#10** | MeSH descriptor: [Movement] this term only |
| **#11** | MeSH descriptor: [Leisure Activities] explode all trees |
| **#12** | MeSH descriptor: [Physical Fitness] explode all trees |
| **#13** | MeSH descriptor: [Walking] explode all trees |
| **#14** | MeSH descriptor: [Yoga] explode all trees |
| **#15** | MeSH descriptor: [Sedentary Behavior] explode all trees |
| **#16** | MeSH descriptor: [Drama] explode all trees |
| **#17** | MeSH descriptor: [Singing] explode all trees |
| **#18** | ("motor activit*" OR "exercis*" OR "sport*" OR "acceleromet*" OR "exertion*" OR "movement*" OR "recreation*" OR "leisure activit*" OR "fitness" OR "walk*" OR "commut*" OR "active transport*" OR "active travel*" OR "active living" OR "active lifestyle*" OR "cycle" OR "cycling" OR "bike*" OR "biking" OR "bicycl*" OR "swim*" OR "yoga" OR "pilates" OR "gardening" OR "running" OR "runner" OR "jogging" OR "danc*" OR "weight lift*" OR "physical train*" OR "exercise train*" OR "strength train*" OR "metabolic equivalent" OR "inactiv*" OR "physical activ*" OR "PAL" OR "workout" OR "MVPA" OR "IPAQ" OR "LTPA" OR "PAEE" OR "sedentar*" OR "screen time" OR "motor behavior*" OR "motor behaviour*" OR "steps" OR "stepping" OR "drama" OR "theatre" OR "theater" OR "actor*" OR "actress*" OR "acting" OR "sing" OR "singing" OR "artistic performance*"):ti,ab,kw |
| **#19** | {OR #6-#18} |
| **Block 3: Determinants/Correlates** | |
|  | ***Generic determinant/correlate terms*** |
| **#20** | ("determinant*" OR "correlat*" OR "influenc*" OR "associat*" OR "facilitat*" OR "barrier*" OR "mediat*" OR "moderat*" OR "contribut*" OR "modifier*" OR "confound*" OR "pattern*" OR "imped*" OR "predict*" OR "factor*" OR "characteristic*" OR "relationship*" OR "motive*" OR "motivator*" OR "psychosocial"):ti,ab,kw |
|  | ***Policy terms*** |
| **#21** | MeSH descriptor: [Public Policy] explode all trees |
| **#22** | ("public polic*" OR "health polic*" OR "road tax*" OR "toll road*" OR "standing break*" OR "workplace*" OR "sport program*" OR "stair design*" OR "physical activity counsel*" OR "workstation" OR "worksite"):ti,ab,kw |
|  | ***Physical environment terms*** |
| **#23** | MeSH descriptor: [Environment Design] explode all trees |
| **#24** | MeSH descriptor: [Residence Characteristics] explode all trees |
| **#25** | MeSH descriptor: [Weather] explode all trees |
| **#26** | MeSH descriptor: [Public Facilities] explode all trees |
| **#27** | MeSH descriptor: [Fitness Centers] explode all trees |
| **#28** | ("environment*" OR "coastal location*" OR "walkab*" OR "aesthetic*" OR "esthetic*" OR "safety" OR "equipment" OR "community design" OR "residence characteristic*" OR "residential density" OR "neighborhood*" OR "neighbourhood*" OR "noise pollution" OR "urban*" OR "rural*" OR "community feature*" OR "community characteristic*" OR "weather" OR "temperature*" OR "winter" OR "season*" OR "precipitation" OR "air pollution" OR "humid*" OR "public facilit*" OR "recreation* facilit*" OR "recreation* location*" OR "recreation* space*" OR "sport* facilit*" OR "facilities" OR "leisure cent*" OR "litter" OR "pathway*" OR "footpath*" OR "sidewalk*" OR "public transport*" OR "public transit" OR "transport environment" OR "service*" OR "shop*" OR "streetlight*" OR "street light*" OR "traffic" OR "gym" OR "gyms" OR "fitness cent*" OR "recreation* cent*" OR "cycle friendly" OR "cycling friendly" OR "park*" OR "pavement*" OR "green space*" OR "blue space*" OR "greenery" OR "open space*" OR "walking trail*" OR "walking path*" OR "walking route*" OR "walking lane*" OR "walking infrastructure" OR "pedestrian trail*" OR "pedestrian path*" OR "pedestrian route*" OR "pedestrian lane*" OR "pedestrian infrastructure" OR "biking trail*" OR "biking path*" OR "biking route*" OR "biking lane*" OR "biking infrastructure" OR "bike trail*" OR "bike path*" OR "bike route*" OR "bike lane*" OR "bike infrastructure" OR "bicycle trail*" OR "bicycle path*" OR "bicycle route*" OR "bicycle lane*" OR "bicycle infrastructure" OR "cycling trail*" OR "cycling path*" OR "cycling route*" OR "cycling lane*" OR "cycling infrastructure" OR "cycle trail*" OR "cycle path*" OR "cycle route*" OR "cycle lane*" OR "cycle infrastructure" OR "road connectivity" OR "street connectivity" OR "land use mix*" OR "distance*" OR "destination*" OR "vandalism" OR "exercis* facilit*"):ti,ab,kw |
|  | ***Interpersonal terms*** |
| **#29** | MeSH descriptor: [Social Environment] explode all trees |
| **#30** | MeSH descriptor: [Crime] explode all trees |
| **#31** | MeSH descriptor: [Culture] explode all trees |
| **#32** | MeSH descriptor: [Social Isolation] explode all trees |
| **#33** | MeSH descriptor: [Communication] explode all trees |
| **#34** | ("social environment*" OR "social connect*" OR "social trust" OR "social cohesion" OR "social participation" OR "social function*" OR "social tie*" OR "community network*" OR "social network*" OR "crime*" OR "criminal*" OR "violen*" OR "social support" OR "family support" OR "peer support" OR "dog owner*" OR "perceived peer attitude*" OR "role model*" OR "parent* physical activ*" OR "parent* activit*" OR "sibling* physical activ*" OR "sibling* activit*" OR "social isolat*" OR "loneliness" OR "social norm*" OR "cultur*" OR "social capital" OR "social programming" OR "communicat*"):ti,ab,kw |
|  | ***Intrapersonal terms*** |
| **#35** | MeSH descriptor: [Personality] explode all trees |
| **#36** | MeSH descriptor: [Motivation] explode all trees |
| **#37** | MeSH descriptor: [Health Status] explode all trees |
| **#38** | MeSH descriptor: [Attitude to Health] explode all trees |
| **#39** | MeSH descriptor: [Health Behavior] explode all trees |
| **#40** | ("self-efficac*" OR "perceived competenc*" OR "intention*" OR "motivation*" OR "enjoyment" OR "planning" OR "action plan*" OR "goal-setting" OR "self-regulat*" OR "habit strength" OR "outcome expecta*" OR "outcome reali*" OR "perception*" OR "preference*" OR "health status" OR "physical health" OR "mental health" OR "wellbeing" OR "well-being" OR "mobilit*" OR "stiff*" OR "physical performance" OR "physical function" OR "knowledge" OR "belief*" OR "attitude*" OR "health behavior*" OR "health behaviour*" OR "personalit*" OR "impulsive*" OR "cognitive abilit*" OR "cognition*" OR "temperament*" OR "perceived behavioral control" OR "perceived behavioural control" OR "perceived physical condition" OR "locus of control" OR "self-esteem" OR "self-concept" OR "self-worth" OR "self-accept*" OR "satisfaction" OR "neurotic*" OR "smok*" OR "alcohol*" OR "food intake" OR "nutritional intake" OR "energy intake" OR "nutrient intake" OR "diet*" OR "stress*" OR "anxiet*" OR "anxious*" OR "depress*" OR "mood*" OR "sleep*" OR "vitality" OR "quality-of-life" OR "pain*" OR "fatigue*" OR "body mass index" OR "BMI" OR "weight status" OR "overweight" OR "underweight" OR "obes*" OR "internet access" OR "internet use" OR "internet usage" OR "health literacy"):ti,ab,kw |
| **#41** | {OR #20-#40} |
| **Block 4: United Kingdom (UK)** | |
|  | ***UK terms*** |
| **#42** | ("uk" OR "u.k." OR "united kingdom" OR "england*" OR "english*" OR "scotland*" OR "scottish*" OR "wales" OR "south wales" OR "welsh*" OR "northern ireland*" OR "northern irish*" OR "gb" OR "g.b." OR "britain*" OR "british*" NOT "new england" NOT "new south wales" NOT "british columbia"):ti,ab,kw |
|  | ***Cross-national terms*** |
| **#43** | ("crosscultural*" OR "cross-cultural*" OR "crossnational*" OR "cross-national*" OR "crossborder*" OR "cross-border*" OR "multinational*" OR "multi-national*" OR "multicultural*" OR "multi-cultural*" OR "international*" OR "transnational*" OR "trans-national*" OR "multicountry" OR "multi-country" OR "multiple countr*" OR "intercountry" OR "inter-country" OR "country comparison*" OR "countries" OR "continent*" OR "intercontinent*" OR "world*" OR "global*" OR "Europe*" OR "Western*" OR "Commonwealth" OR "cohorts" OR "studies" OR "harmoni*"):ti |
| **#44** | #42 OR #43 |
| **Block 5: Socio-economic status** | |
|  | ***Socio-economic status terms*** |
| **#45** | MeSH descriptor: [Socioeconomic Factors] explode all trees |
| **#46** | ("occupation*" OR "income" OR "educat*" OR "socioeconomic*" OR "socio-economic*" OR "social class*" OR "ses" OR "disadvantag*" OR "underserved" OR "privilege*" OR "depriv*" OR "poverty" OR "inequalit*" OR "low socio*" OR "high socio*" OR "poverty area*" OR "IMD" OR "dispar*" OR "employ*"):ti,ab,kw |
| **#47** | #45 OR #46 |
| **Proximity operators: Combining searches** | |
| **#48** | #5 AND #19 AND #41 AND #44 AND #47 |

*Captures alternative word endings

*MeSH*, Medical Subject Headings

**Table S5.** Scopus search strategy

| **Block 1: Population** | |
| --- | --- |
| **1** | ***Population terms*** |
|  | TITLE-ABS-KEY ( "old* age*" OR "aging" OR "ageing" OR "middle age*" OR "older adult*" OR "older person*" OR "older people*" OR "elder*" OR "senior" OR "seniors" OR "geriatric*" AND NOT ( "child*" AND NOT "adult*" ) AND NOT ("adolescen*" NOT "adult*" )) |
| **Block 2 : Outcome** | |
| **2** | ***Outcome terms*** |
|  | TITLE-ABS-KEY ( "motor activit*" OR "exercis*" OR "sport*" OR "acceleromet*" OR "exertion*" OR "movement*" OR "recreation*" OR "leisure activit*" OR "fitness" OR "walk*" OR "commut*" OR "active transport*" OR "active travel*" OR "active living" OR "active lifestyle*" OR "cycle" OR "cycling" OR "bike*" OR "biking" OR "bicycl*" OR "swim*" OR "yoga" OR "pilates" OR "gardening" OR "running" OR "runner" OR "jogging" OR "danc*" OR "weight lift*" OR "physical train*" OR "exercise train*" OR "strength train*" OR "metabolic equivalent" OR "inactiv*" OR "physical activ*" OR "pal" OR "workout" OR "mvpa" OR "ipaq" OR "ltpa" OR "paee" OR "sedentar*" OR "screen time" OR "motor behavior*" OR "motor behaviour*" OR "steps" OR "stepping" OR "drama" OR "theatre" OR "theater" OR "actor*" OR "actress*" OR "acting" OR "sing" OR "singing" OR "artistic performance*" ) |
| **Block 3: Determinants/Correlates** | |
| **3** | ***Generic determinant/correlate terms*** |
|  | TITLE-ABS-KEY ( "determinant*" OR "correlat*" OR "influenc*" OR "associat*" OR "facilitat*" OR "barrier*" OR "mediat*" OR "moderat*" OR "contribut*" OR "modifier*" OR "confound*" OR "pattern*" OR "imped*" OR "predict*" OR "factor*" OR "characteristic*" OR "relationship*" OR "motive*" OR "motivator*" OR "psychosocial" ) |
| **4** | ***Policy terms*** |
|  | TITLE-ABS-KEY ( "public polic*" OR "health polic*" OR "road tax*" OR "toll road*" OR "standing break*" OR "workplace*" OR "sport program*" OR "stair design*" OR "physical activity counsel*" OR "workstation" OR "worksite" ) |
| **5** | ***Physical environment terms*** |
|  | TITLE-ABS-KEY ( "environment*" OR "coastal location*" OR "walkab*" OR "aesthetic*" OR "esthetic*" OR "safety" OR "equipment" OR "community design" OR "residence characteristic*" OR "residential density" OR "neighborhood*" OR "neighbourhood*" OR "noise pollution" OR "urban*" OR "rural*" OR "community feature*" OR "community characteristic*" OR "weather" OR "temperature*" OR "winter" OR "season*" OR "precipitation" OR "air pollution" OR "humid*" OR "public facilit*" OR "recreation* facilit*" OR "recreation* location*" OR "recreation* space*" OR "sport* facilit*" OR "facilities" OR "leisure cent*" OR "litter" OR "pathway*" OR "footpath*" OR "sidewalk*" OR "public transport*" OR "public transit" OR "transport environment" OR "service*" OR "shop*" OR "streetlight*" OR "street light*" OR "traffic" OR "gym" OR "gyms" OR "fitness cent*" OR "recreation* cent*" OR "cycle friendly" OR "cycling friendly" OR "park*" OR "pavement*" OR "green space*" OR "blue space*" OR "greenery" OR "open space*" OR "walking trail*" OR "walking path*" OR "walking route*" OR "walking lane*" OR "walking infrastructure" OR "pedestrian trail*" OR "pedestrian path*" OR "pedestrian route*" OR "pedestrian lane*" OR "pedestrian infrastructure" OR "biking trail*" OR "biking path*" OR "biking route*" OR "biking lane*" OR "biking infrastructure" OR "bike trail*" OR "bike path*" OR "bike route*" OR "bike lane*" OR "bike infrastructure" OR "bicycle trail*" OR "bicycle path*" OR "bicycle route*" OR "bicycle lane*" OR "bicycle infrastructure" OR "cycling trail*" OR "cycling path*" OR "cycling route*" OR "cycling lane*" OR "cycling infrastructure" OR "cycle trail*" OR "cycle path*" OR "cycle route*" OR "cycle lane*" OR "cycle infrastructure" OR "road connectivity" OR "street connectivity" OR "land use mix*" OR "distance*" OR "destination*" OR "vandalism" OR "exercis* facilit*" ) |
| **6** | ***Interpersonal terms*** |
|  | TITLE-ABS-KEY ( "social environment*" OR "social connect*" OR "social trust" OR "social cohesion" OR "social participation" OR "social function*" OR "social tie*" OR "community network*" OR "social network*" OR "crime*" OR "criminal*" OR "violen*" OR "social support" OR "family support" OR "peer support" OR "dog owner*" OR "perceived peer attitude*" OR "role model*" OR "parent* physical activ*" OR "parent* activit*" OR "sibling* physical activ*" OR "sibling* activit*" OR "social isolat*" OR "loneliness" OR "social norm*" OR "cultur*" OR "social capital" OR "social programming" OR "communicat*" ) |
| **7** | ***Intrapersonal terms*** |
|  | TITLE-ABS-KEY ( "self-efficac*" OR "perceived competenc*" OR "intention*" OR "motivation*" OR "enjoyment" OR "planning" OR "action plan*" OR "goal-setting" OR "self-regulat*" OR "habit strength" OR "outcome expecta*" OR "outcome reali*" OR "perception*" OR "preference*" OR "health status" OR "physical health" OR "mental health" OR "wellbeing" OR "well-being" OR "mobilit*" OR "stiff*" OR "physical performance" OR "physical function" OR "knowledge" OR "belief*" OR "attitude*" OR "health behavior*" OR "health behaviour*" OR "personalit*" OR "impulsive*" OR "cognitive abilit*" OR "cognition*" OR "temperament*" OR "perceived behavioral control" OR "perceived behavioural control" OR "perceived physical condition" OR "locus of control" OR "self-esteem" OR "self-concept" OR "self-worth" OR "self-accept*" OR "satisfaction" OR "neurotic*" OR "smok*" OR "alcohol*" OR "food intake" OR "nutritional intake" OR "energy intake" OR "nutrient intake" OR "diet*" OR "stress*" OR "anxiet*" OR "anxious*" OR "depress*" OR "mood*" OR "sleep*" OR "vitality" OR "quality-of-life" OR "pain*" OR "fatigue*" OR "body mass index" OR "bmi" OR "weight status" OR "overweight" OR "underweight" OR "obes*" OR "internet access" OR "internet use" OR "internet usage" OR "health literacy" ) |
| **Block 4: United Kingdom (UK)** | |
| **8** | ***UK terms*** |
|  | TITLE-ABS-KEY ( "uk" OR "u.k." OR "united kingdom" OR "england*" OR "english*" OR "scotland*" OR "scottish*" OR "wales" OR "south wales" OR "welsh*" OR "northern ireland*" OR "northern irish*" OR "gb" OR "g.b." OR "britain*" OR "british*" AND NOT "new england" AND NOT "new south wales" AND NOT "british columbia" ) |
| **9** | ***Cross-national terms*** |
|  | TITLE ( "crosscultural*" OR "cross-cultural*" OR "crossnational*" OR "cross-national*" OR "crossborder*" OR "cross-border*" OR "multinational*" OR "multi-national*" OR "multicultural*" OR "multi-cultural*" OR "international*" OR "transnational*" OR "trans-national*" OR "multicountry" OR "multi-country" OR "multiple countr*" OR "intercountry" OR "inter-country" OR "country comparison*" OR {countries} OR "continent*" OR "intercontinent*" OR "world*" OR "global*" OR "europe*" OR "western*" OR "commonwealth" OR {cohorts} OR {studies} OR "harmoni*" ) |
| **Block 5: Filters** | |
| **10** | ***Filters*** |
|  | ( EXCLUDE ( DOCTYPE , "re" ) OR EXCLUDE ( DOCTYPE , "ch" ) OR EXCLUDE ( DOCTYPE , "bk" ) OR EXCLUDE ( DOCTYPE , "ed" ) ) AND ( LIMIT-TO ( LANGUAGE , "english" ) ) |
| **Block 6: Socio-economic status** | |
| **11** | ***Socio-economic status terms*** |
|  | TITLE-ABS-KEY ( "occupation*" OR "income" OR "educat*" OR "socioeconomic*" OR "socio-economic*" OR "social class*" OR "ses" OR "disadvantag*" OR "underserved" OR "privilege*" OR "depriv*" OR "poverty" OR "inequalit*" OR "low socio*" OR "high socio*" OR "poverty area*" OR "imd" OR "dispar*" OR "employ*" ) |
| **Proximity operators: Combining searches** | |
| **12** | (3 OR 4 OR 5 OR 6 OR 7) |
| **13** | (8 OR 9) |
| **14** | 1 AND 2 AND 10 AND 11 AND 12 AND 13 |

*Captures alternative word endings
