# Supplementary material for: Correlates and determinants of physical activity among older adults of lower versus higher socio-economic status: a systematic review and meta-analysis
Source: Int J Behav Nutr Phys Act. 2025 Jun 23;22:83. doi: 10.1186/s12966-025-01775-y (PMC12183859; doi:10.1186/s12966-025-01775-y)
Supplement: Supplementary file 4 — Additional file 4. Mixed Methods Appraisal Tool (MMAT) guidance notes. [file 12966_2025_1775_MOESM4_ESM.docx]

**Additional file 4.** Mixed Methods Appraisal Tool (MMAT) guidance notes

Ideally, even if you rate “Can’t tell” to begin with, you will find additional information (e.g., in another published study or protocol using the same data) that will allow you to change this response to “Yes” or “No”.

**Screening questions:**

*S1. Are there clear research questions?*

- See MMAT user guide page 1.

*S2. Do the collected data allow to address the research questions?*

- See MMAT user guide page 1.

**2. Quantitative randomised controlled trials:**

*2.1. Is randomization appropriately performed?*

- See MMAT methodological quality criterion (appropriate randomisation, e.g., random number generator, and allocation concealment)
- To answer “Yes”, both the randomisation procedure (e.g., random number generator), and allocation concealment (i.e., both the researchers and participants should be unaware of the assignment sequence until allocation) conditions must be met

*2.2. Are the groups comparable at baseline?*

- Focus specifically on whether any observed differences at baseline would threaten the validity of the study (e.g., make it challenging to attribute any effects to the intervention itself, over and above other factors)
- If participant numbers are small, baseline characteristics would be expected to differ slightly across groups (this is acceptable, if there are no major differences in the outcome variable at baseline)

*2.3. Are there complete outcome data?*

- The following thresholds have been chosen: > 80% complete data for cross-sectional studies, and less than 20% withdrawal/dropout acceptable for follow-up of more than a year (among those meeting the original study’s inclusion criteria)
- Look at proportions of missing data across variables (if not presented in the publication but OSM has access to the dataset and/or extracted supplementary data, OSM will check this), and for longitudinal studies, across timepoints
- Focus on outcome data, rather than other variables

*2.4. Are outcome assessors blinded to the intervention provided?*

- See MMAT methodological quality criterion
- Rate “No” if participants and outcome assessors were not blind to the treatment allocation

*2.5. Did the participants adhere to the assigned intervention?*

- See MMAT methodological quality criterion
- Rate “Yes” if at least 80% of participants attended all intervention/programme sessions (or complied with procedures, or received the intervention as per protocol)
- Rate “No” if high crossover rate reported

**3. Quantitative non-randomised:**

*3.1. Are the participants representative of the target population?*

- Evidence of a representative sample (among the population sub-group that was targeted in the study’s inclusion/exclusion criteria), particularly in relation to age, sex/gender
- For cohort studies (typically > 2,000 participants), rate “Yes” if analyses were weighted to calibrate back to population distributions (particularly for age and sex – within the 60+ years age group specifically)
- For cohort studies, rate “No” if analyses are not weighted (the publication may also present evidence of differences in the characteristics of included and excluded participants)
- For smaller non-cohort studies, if the analyses are not weighted, but the sample is designed to be representative (e.g., efforts to match proportion of males/females to national proportions, survey quotas, probability sampling, simple random sampling, or non-probability sampling), that is fine, and you can rate “Yes”
- If it is a standalone study, and there is no information on representativeness (and there are no other papers describing the sample), you can rate “No” rather than “Can’t tell”

*3.2. Are measurements appropriate regarding both the outcome and intervention (or exposure)?*

- Base ratings on the main exposure(s) and outcome(s), rather than covariates or secondary outcomes etc.
- If device-based measures exist (e.g., for physical activity), and they were not used for the exposure and/or outcome, rate “No”
- If device-based measures were used where they exist, and/or validated self-report measures were used where no device-based alternatives exist, rate “Yes”
- If the main exposure of interest is demographic, that is fine (the item/scale does not have to be validated), you can rate “Yes” if the main outcome variable(s) is device-measured (and/or using a validated self-report measure if no device-based measures exist)

*3.3. Are there complete outcome data?*

- The following thresholds have been chosen: > 80% complete data for cross-sectional studies, and less than 20% withdrawal/dropout acceptable for follow-up of more than a year (among those meeting the original study’s inclusion criteria)
- Look at proportions of missing data across variables (if not presented in the publication but OSM has access to the dataset and/or extracted supplementary data, OSM will check this), and for longitudinal studies, across timepoints
- Focus on outcome data, rather than other variables

*3.4. Are the confounders accounted for in the design and analysis?*

- Adjustment for key covariates or stratification/matching/standardisation/inverse probability weighting – mark “Yes” if statistical analyses adjusted for at least age, sex, and one socio-economic measure, for at least one of the outcome measures (if the analyses are stratified by sex, and adjusted for age and one socio-economic measure, that is acceptable – if the population is 100% male or female, that is fine too, as there would be no reason to adjust for sex)
- If the population was limited to a small age group e.g., range of 10 or less years (60–69 years) and therefore age wasn’t controlled for, that is fine
- Acceptable if included those variables as covariates to begin with, but they were then removed statistically (e.g., because of backwards elimination or stepwise regression)
- If a measure of socio-economic status is the main exposure of interest (rather than adjusted for as a covariate), that is fine

*3.5. During the study period, is the intervention administered (or exposure occurred) as intended?*

- For intervention studies, please see the MMAT user guide
- To simplify things, for observational studies, rate “Yes” if the study explored how changes in the exposure were associated with changes in the outcome (this type of analysis fully exploits the benefits of a longitudinal design)
- Rate “Yes” if both the main exposure of interest and the outcome were assessed at ≥ 2 timepoints (and data for both or more timepoints were included in analyses for each of the exposure and outcome variables)
- Rate “Yes” if the exposure was assessed at ≥ 2 timepoints and the outcome was only assessed at a singular follow-up timepoint (or vice versa) – one or both of the main exposure and outcome of interest need to be assessed at ≥ 2 timepoints (i.e., need to be included in analyses as time-varying)
- Rate “No” if the study was longitudinal, but assessed each of the exposure and the outcome measures at a single point in time (e.g., exposure assessed at time 1, and outcome assessed at time 2) – exceptions to the rule: you can rate “Yes” if the analysis controlled for the exposure/outcome at a previous timepoint or if the main exposure of interest is demographic or geographic (and therefore not modifiable, at least over a short timescale, so there would be no reason to explore “changes” in the exposure)
- Rate “No” if the study was cross-sectional (or repeated cross-sectional)

**4. Quantitative descriptive:**

*4.1. Is the sampling strategy relevant to address the research question?*

- See MMAT methodological quality criterion
- Focus on appropriateness of sampling procedure for target population (and clear justification provided)

*4.2. Is the sample representative of the target population?*

- Evidence of a representative sample (among the population sub-group that was targeted in the study’s inclusion/exclusion criteria), particularly in relation to age, sex/gender
- For smaller non-cohort studies, if the analyses are not weighted, but the sample is designed to be representative (e.g., efforts to match proportion of males/females to national proportions, survey quotas, probability sampling, simple random sampling, or non-probability sampling), that is fine, and you can rate “Yes”
- If it is a standalone study, and there is no information on representativeness (and there are no other papers describing the sample), you can rate “No” rather than “Can’t tell”

*4.3. Are the measurements appropriate?*

- Base ratings on the main variables
- If device-based measures exist, and they were not used, rate “No”
- If device-based measures were used where they exist, and/or validated self-report measures were used where no device-based alternatives exist, rate “Yes”
- If a variable is demographic, that is fine (the item/scale does not have to be validated), you can rate “Yes” if the other main variable(s) is device-measured (and/or using a validated self-report measure if no device-based measures exist)
- If there is no mention of validity/reliability (and this information is not available elsewhere), and the questions have not been taken from other surveys (e.g., no citation), you can rate “No”

*4.4. Is the risk of nonresponse bias low?*

- Focus specifically on whether there are any observed differences between respondents and non-respondents (this can be by area – e.g., more respondents from wealthier areas – if individual participant comparisons are not presented)

*4.5. Is the statistical analysis appropriate to answer the research question?*

- Appropriate for research question (descriptive statistics)

**5. Mixed methods:**

*5.1. Is there an adequate rationale for using a mixed methods design to address the research question?*

- See MMAT methodological quality criterion
- Rate “No” if no rationale for conducting a mixed methods study is given

*5.2. Are the different components of the study effectively integrated to answer the research question?*

- See MMAT methodological quality criterion
- How and when integration occurred
- If data collection, analysis, and interpretation are conducted separately (i.e., no interrelations, connections, or comparisons between quantitative and qualitative data), rate “No”

*5.3. Are the outputs of the integration of qualitative and quantitative components adequately interpreted?*

- Integration in the interpretation of the quantitative and qualitative components (the results of each component add value to one another above and beyond conducting two separate studies)
- If results presented and discussed separately, rate “No”

*5.4. Are divergences and inconsistencies between quantitative and qualitative results adequately addressed?*

- Rate “Yes” if there is no divergence
- Rate “Yes” if divergences are explained
- Rate “No” if results presented and discussed separately

*5.5. Do the different components of the study adhere to the quality criteria of each tradition of the methods involved?*

- See MMAT methodological quality criterion
- Rate the quantitative (2.1–2.5, 3.1–3.5, or 4.1–4.5) and qualitative (1.1–1.5) components individually based on the relevant criteria
- Choose the weakest of the quantitative and qualitative components (i.e., the component with the least “Yes” ratings or the most “No” ratings out of the five criteria for that study design)
- Based on the weakest component (quantitative or qualitative), rate “No” to 5.5 if there were < 4 “Yes” ratings and rate “Yes” if there were ≥ 4 “Yes” ratings
