# Supplementary material for: Correlates and determinants of physical activity among older adults of lower versus higher socio-economic status: a systematic review and meta-analysis
Source: Int J Behav Nutr Phys Act. 2025 Jun 23;22:83. doi: 10.1186/s12966-025-01775-y (PMC12183859; doi:10.1186/s12966-025-01775-y)
Supplement: Supplementary file 5 — Additional file 5. Characteristics of the included studies. [file 12966_2025_1775_MOESM5_ESM.docx]

**Additional file 5.** Characteristics of the included studies

| **Author(s) and year**  **Country**  **Dataset**  **Waves in study** | **Study design (follow-up)**  **Funding source(s)**  **Waves in review** | **Sample characteristics** | **Perspective or theoretical framework** | **Physical activity** | **Physical activity measure quality^a^** | | | | | | | | | **Independent variables (name of exposure if included in meta-analyses)** |
| --- | --- | --- | --- | --- | --- | --- | --- | --- | --- | --- | --- | --- | --- | --- |
|  |  |  |  |  | 1 | 2 | 3 | 4 | 5 | 6 | 7 | 8 | 9 |  |
| Alkhatib, 2013 [28]^*^  England | Cross-sectional; quantitative non-randomised  Not reported | 34 participants (mean age: 47.8 ± 11.9 years); 15 male, 19 female  *Sub-sample:*  5 participants aged 60+ years; 3 male, 2 female  SES (job role):  Lower (manual): 1  Higher (non-manual): 4 | Not stated | Measure: International Physical Activity Questionnaire  Outcome in review: Dichotomous | + | ? | + | + | + |  | - | + | + | - Glucose  - Cholesterol  - Dominant hand grip strength  - Non-dominant hand grip strength  - Systolic blood pressure  - Diastolic blood pressure  - Flexibility (physical function)  - V̇O_2_ max  - Body mass index (weight status)  - Fat % |
| Allen et al., 2019 [29]^†^  England  English Longitudinal Study of Ageing  Wave 5, 6 | Prospective; quantitative non-randomised (2 years)  Not applicable  Wave 5 | 8,476 participants (mean age: 67.42 ± 8.80 years); 3,804 male, 4,572 female  *Sub-sample:*  6,183 community-dwelling participants aged 60+ years; 2,745 male, 3,438 female  SES (age finished continuous full-time education):  Lower (never went to school/14 or under/age 15/age 16/age 17): 4,743  Higher (not yet finished/age 18/19 or over): 1,436 | Not stated | Outcome in review: Dichotomous and continuous | ? | ? | - | + | - |  | - | + | + | - Television viewing  - Alcohol involvement (alcohol consumption)  - Diet (fruit and vegetable consumption) |
| Anokye et al., 2013 [30]^†^  England  Health Survey for England 2006 | Cross-sectional; quantitative non-randomised  Department of Health’s Policy Research Programme  2006 | 14,142 participants (mean age: 49.3 ± 18.6 years); 6,324 male, 7,818 female  *Sub-sample:*  4,381 participants aged 60+ years; 1,961 male, 2,420 female  SES (equivalised income, divided into quintiles):  Lower (quintiles 1 and 2): 1,491  Higher (quintiles 3 to 5): 1,792 | Not stated | Outcome in review: Dichotomous | ? | ? | ? | + | + |  | ? | + | + | - Drinkers (alcohol consumption)  - Voluntary activities |
| Barnett et al., 2014 [31]^‡^  England  European Prospective Investigation into Cancer and Nutrition (EPIC)–Norfolk  Baseline: 1997–2000  Follow-up 1: 2002–2006  Follow-up 2: 2006–2007 | Prospective; quantitative non-randomised (up to 10 years)  British Heart Foundation  Economic and Social Research Council  Medical Research Council  National Institute for Health Research  Wellcome Trust  Baseline and follow-up 2 | 3,334 participants (mean age: employed to retired, 59.7 ± 4.7 years; remained employed, 53.0 ± 5.1 years); 1,600 male, 1,734 female  SES (social class):  Lower (manual): 1,219  Higher (non-manual): 2,115  *Sub-sample:*  599 participants aged 60+ years  SES (social class):  Lower (manual): 253  Higher (non-manual): 346 | Not stated | Measure:  EPIC Physical Activity Questionnaire (EPAQ2)  Outcome in review: Continuous | + | ? | + |  | + |  | - | + | + | - Body mass index, covariate (weight status)  - Smoking status, covariate (smoking status)  - Alcohol consumption, covariate (alcohol consumption) |
| Bennie et al., 2020 [32]^†^  UK  European Health Interview Survey  Wave 2 | Cross-sectional; quantitative non-randomised  Not applicable  Wave 2 | 280,605 participants (age range: 18 to 75+ years; 20,161 UK participants); 127,075 male, 153,530 female  SES (equivalised household income quintiles):  Lower (1^st^ and 2^nd^ quintile): 103,199  Higher (3^rd^ to 5^th^ quintile): 160,315  *Sub-sample:*  8,630 UK participants aged 60+ years with available physical activity data; 3,981 male, 4,649 female  SES (equivalised household income quintiles):  Lower (below 1^st^ quintile/between 1^st^ quintile and 2^nd^ quintile): 3,660  Higher (between 2^nd^ quintile and 5^th^ quintile): 4,968 | Not stated | Measure: European Health Interview Survey Physical Activity Questionnaire  Outcome in review: Dichotomous | ? | ? | - | + | - |  | ? | + | + | - Self-rated health (perception of general health)  - Body mass index (weight status) |
| Berger et al., 2005 [79]^∮^  Scotland  West of Scotland Twenty-07 ‘Health in the Community’ study  1991 and 1995/96 | Prospective; quantitative non-randomised (4–5 years)  Not reported  1991 and 1995/96 | 699 participants (age range: ~60 to ~64 years); 54.5% female  SES (social class):  Lower (manual): 55.4% in 1991, 56.2% in 1995/96  Higher (non-manual): 44.6% in 1991, 43.8% in 1995/96 | Not stated | Outcome in review: Dichotomous | ? | ? | ? | + | + |  | ? | + | + | - Smoking  - Healthiness of diet  - Self-rated health  - Disability |
| Bourassa et al., 2019 [80]^‡^  England  English Longitudinal Study of Ageing  Wave 2, 3, 4, 5, 6 | Prospective; quantitative non-randomised (4 years)  National Institute of Child Health & Human Development  Wave 2, 3 | 5,786 participants (mean age: 63.14 ± 8.99 years); 53.9% female  *Sub-sample:*  3,439 participants aged 60+ years (mean age: 68.9 ± 6.6 years); 1,729 female  SES (household wealth):  Lower (below or equal to median): 1,744  Higher (strictly above median): 1,695 | Not stated | Outcome in review: Continuous | ? | ? | - | + | - |  | - | + | + | - Life satisfaction |
| Brainard et al., 2019 [33]^†^  England  Adult Active Lives Survey 2016/17 | Cross-sectional; quantitative non-randomised  Sport England  2016/17 | 194,756 participants (age range: 16 to 104 years); 55.8% female  *Sub-sample:*  75,716 participants aged 60+ years; 36,885 male, 38,831 female  SES (IMD 2015 deciles):  Lower (deciles 6–10): 33,445  Higher (deciles 1–5): 42,271 | Not stated | Outcome in review: Dichotomous and continuous | ? | ? | ? | + | + |  | ? | + | + | - Happy (depressive symptoms)  - Anxious  - Worthwhile  - Life satisfaction  - Can’t achieve goals |
| Burnett et al., 2021 [34]^‡^  UK | Cross-sectional; quantitative non-randomised  Medical Research Council  Chief Scientist Office Medical Research Council and University of Glasgow College of Medical, Veterinary and Life Sciences PhD studentship | 2,252 participants (age range: 18 to 65+ years); 1,018 male, 1,234 female  SES (social grade):  Lower (manual, C2DE): 833  Higher (non-manual, ABC1): 1,419  *Sub-sample:*  222 participants aged 65+ years  SES (social grade):  Lower (manual, C2DE): 98  Higher (non-manual, ABC1): 124 | Not stated | Outcome in review: Dichotomous | ? | ? | ? | + | - |  | ? | + | + | - Dog ownership (dog ownership) |
| Cajita et al., 2017 [35]^‡^  UK  Building Research Initiative Group: Chronic Illness Management and Adherence in Transplantation (BRIGHT) study | Cross-sectional; quantitative non-randomised  National Institute of Nursing Research Ruth L. Kirschstein National Research Service Award | 1,379 participants (mean age: 53.5 ± 13.0 years; age range: 18.3 to 80.8 years; 99 UK participants); 987 male, 373 female  SES (educational attainment):  Lower (primary/secondary): 602  Higher (University): 746  *Sub-sample:*  29 UK participants aged 60+ years  SES (educational attainment):  Lower (primary/secondary): 16  Higher (University): 13 | Health Literacy and Health Outcomes Framework | Measure: 2-item Brief Physical Activity Assessment Tool  Outcome in review: Dichotomous | + | ? | - | + | + |  | - | + | + | - Health literacy (health literacy)  - Received advice from healthcare provider, covariate (social support)  - Depressive symptoms, covariate (depressive symptoms) |
| Chaudhury and Shelton, 2010 [81]^†^  England  Health Survey for England  2006, 2007 | Cross-sectional; quantitative non-randomised  Not reported  2006, 2007 | 1,550 participants in 2006 (age range: 60 to 69 years); 715 male, 834 female  SES (equivalised household income quintiles):  Lower (4^th^ and 5^th^ quintiles): 473  Higher (1^st^ to 3^rd^ quintiles): 1,080  561 participants in 2007 (age range: 60 to 64 years); 244 male, 317 female  SES (equivalised household income quintiles):  Lower (4^th^ and 5^th^ quintiles): 159  Higher (1^st^ to 3^rd^ quintiles): 402 | Not stated | Outcome in review: Dichotomous | ? | ? | ? | + | + |  | ? | + | + | - Waist circumference  - Waist-to-hip ratio  Barriers, stops from more physical activity:  - Don’t need to do more  - Work commitments  - Don’t have enough leisure time  - Caring for children or older people  - Have no one to exercise with  - No suitable places to do it in area  - Haven’t got the right clothes or equipment  - Poor health or physical limitations  Barriers, other factors prevent physical activity:  - Not the sporty type  - Too shy or embarrassed  - Worried about injury  - Too old  - Prefer to do other things  - Think exercise is a waste of time  - Too overweight  - Not motivated to do more  - Don’t enjoy physical activity |
| Cholerton et al., 2022 [82]^∮^  UK | Cross-sectional; quantitative non-randomised  PhD funding from the Academy of Sport and Physical Activity, Sheffield Hallam University | 439 participants (age range: 50 to 75 years); 392 male, 47 female  SES (IMD rankings, divided into two groups):  Lower (more deprived areas): 142  Higher (less deprived areas): 297 | Not stated | Not applicable |  |  |  |  |  |  |  |  |  | Influences on walking football initiation:  - Psychological influences  - Social interactions  - Walking football environment  Influences on walking football maintenance:  - Psychological influences  - Social-level influences  - Walking football culture  - Walking football session specific factors  - Availability of maintenance resources |
| Cook, 2021 [83]^‡^  UK | Cross-sectional; quantitative descriptive  Economic and Social Research Council | 287 participants (age range: 18 to 64 years); 62.46% male, 37.64% female  *Sub-sample:*  2 participants aged 60+ years  SES (household income):  Lower (less than £25,000): 1  Higher (£25,000 or above): 1 | The Production of Mobilities Mesotheory | Not applicable |  |  |  |  |  |  |  |  |  | Factors affecting decision to run commute on any given day:  - Air pollution  - Facilities at different work locations  - Others running in too  - Formal dress requirements  - Already prepped running gear  - Organised/incentives to run  - Other commitments – social, family, personal  - What needs to be carried  - Work hours/commitments/locations  - Weather and climate, seasonality  - Mood  - Logistics/feasibility with other commutes  - Running/fitness schedule  - Running abilities/injuries/energy levels/health  - Location of clothes/objects |
| Coronini-Cronberg et al., 2012 [84]^∮^  England  National Travel Survey  2005, 2006, 2007, 2008 | Repeated cross-sectional; quantitative non-randomised  National Institute for Health Research Collaboration for Leadership in Applied Health Research & Care scheme  National Institute for Health Research Biomedical Research Centre scheme  Imperial Centre for Patient Safety and Service Quality  Higher Education Funding  Council for England  National Institute for Health Research  London Deanery  2005, 2006, 2007, 2008 | 16,911 participants (age range: ≥ 60 years); 7,787 male, 9,124 female  SES (housing tenure):  Lower (rented): 3,275  Higher (owned): 13,636 | Not stated | Outcome in review: Dichotomous | ? | ? | ? | + | - |  | ? | + | + | - Bus pass holder |
| Daly et al., 2019 [85]^‡^  England  English Longitudinal Study of Ageing  Wave 1 (2002/2003) to 2012 | Prospective; quantitative non-randomised (9–10 years)  Economic and Social Research Council  European Union’s H2020 Work Programme (2014–2020)  Wave 1 | 9,949 participants (mean age: 64.88 ± 10.50 years); 54.15% female  9,604 participants aged 60+ years  SES (wealth deciles):  Lower (below or equal to the median): 4,694  Higher (strictly above the median): 4,910 | Not stated | Outcome in review: Continuous | ? | ? | - | + | - |  | - | + | + | - Time perspective |
| Dawson et al., 2007 [86]^∮^  England, Scotland | Cross-sectional; quantitative non-randomised  The Countryside Agency  British Heart Foundation  New Opportunities Scheme | 680 participants (mean age: 66.6 ± 7.5 years; age range: 50 to 93 years); 492 female  SES (IMD 2004 quintiles):  1–most affluent: 130  2: 141  3: 135  4: 134  5–most deprived: 129 | Ecological framework | Not applicable |  |  |  |  |  |  |  |  |  | Perceived barriers to walking in the neighbourhood:  - Health problem  - No one to walk with  - Nowhere green or pleasant  - No pavement  - Nowhere to go, e.g., shop, pub, church  - Personal-safety worries  - Worry about cyclists on pavement  - Tripping over broken paving stones  - Too much traffic  - Too much pollution |
| Dėdelė et al., 2022 [87]^†^  UK  Eurobarometer 88.4  2017 | Cross-sectional; quantitative non-randomised  Not applicable  2017 | 28,031 participants (mean age: 48.3 ± 18.8 years); 51.8% female  *Sub-sample:*  Of the 1,338 UK participants, 520 aged 60+ years; 275 male, 245 female  SES (household income quintiles):  Lower (1^st^ and 2^nd^ quintiles): 229  Higher (3^rd^ to 5^th^ quintiles): 88 | Self-determination theory | Outcome in review: Dichotomous | ? | ? | ? | + | + |  | ? | + | + | Positive:  - Health improvement  - Physical appearance  - Counteract aging  - Have fun  - Relax  - Be with friends  - Make acquaintances  - Meet other cultures  - Physical performance  - Fitness  - Control weight  - Self-esteem  - New skills  - Spirit of competition  - Social integration    Negative:  - No time  - Competitivity  - No infrastructure  - Disability/illness  - No friends  - Feel discriminated  - Lack motivation  - Risk of injuries |
| Ding and Stamatakis, 2014 [36]^*^  England  Health Survey for England  Time point 1: 1997–1999  Time point 2: 2003–2004  Time point 3: 2006/08 | Repeated cross-sectional; quantitative non-randomised  Australian National Health and Medical Research Council  National Institute for Health Research  Time point 1, 2, and 3 | 81,090 participants (mean age, 95% CI: 46.7, 45.5–47.9 years (yoga practice: yes); 46.5, 46.5–46.8 years (yoga practice: no)); 88.4% female (yoga practice: yes); 54.8% female (yoga practice: no)  SES (tertiary degree or higher):  Lower (no): 67.2% (yoga practice: yes); 85.1% (yoga practice: no)  Higher (yes): 32.8% (yoga practice: yes); 14.9% (yoga practice: no)  *Sub-sample:*  21,674 participants aged 60+ years; 9,641 male, 12,033 female  SES (tertiary degree or higher):  Lower (no): 20,101  Higher (yes): 1,520 | Not stated | Outcome in review: Dichotomous | ? | ? | ? | + | - |  | ? | + | + | - Depression (depressive symptoms) |
| Ding et al., 2017 [37]^†^  England  English Longitudinal Study of Ageing  Wave 2, 3, 4, 6 | Prospective; quantitative non-randomised (8 years)  Not reported  Wave 2, 4 | 4,638 participants (mean age: 74.0 ± 6.3 years; age range: 65 to 89 years); 2,070 male, 2,568 female  SES (deciles of non-pension wealth):  Lower (lowest 2 deciles): 980  Higher (highest 8 deciles): 3,577  *Sub-sample:*  4,597 community-dwelling participants aged 60+ years; 2,047 male, 2,550 female  SES (deciles of non-pension wealth):  Lower (lowest 2 deciles): 976  Higher (highest 8 deciles): 3,581 | Working framework of the Canadian Initiative for Frailty and Aging | Outcome in review: Dichotomous and continuous | ? | ? | - | + | - |  | - | + | + | - Slowness: gait speed (physical function)  - Weakness: dominant hand grip strength |
| Elliott et al., 2023 [38]^†^  UK  BlueHealth International Survey  2017–2018 | Cross-sectional; quantitative non-randomised  European Union’s Horizon 2020 research and innovation programme  2017–2018 | 15,917 participants (age range: ≥ 18 years); 51% female  *Sub-sample:*  Of the 1,119 UK participants, 419 aged 60+ years; 191 male, 228 female  SES (reported comfort with current household income):  Lower (finding it difficult/very difficult): 52  Higher (coping/living comfortably): 366 | Nature and health theoretical framework | Outcome in review: Dichotomous and continuous | + | ? | + |  | + |  | ? | + | + | - Green space (amount of green space)  - Inland blue space  - Coastal blue space  - Green space visits  - Inland blue space visits  - Coastal blue space visits |
| Flowers et al., 2016 [39]^†^  UK | Cross-sectional; quantitative non-randomised  Economic and Social Research Council | 1,988 participants (mean age: 43.19 ± 11.46 years; age range: 22 to 65 years); 997 male, 991 female  *Sub-sample:*  194 participants aged 60+ years; 112 male, 82 female  SES (income category):  Lower (less than £25,000): 55  Higher (£25,000 or more): 107 | Schematic model of motivational processes underlying the relationship between natural environments and physical activity behaviours | Measure: Short form of the International Physical Activity Questionnaire  Outcome in review: Dichotomous | + | ? | + | + | + |  | - | + | + | - Self-reported health (perception of general health)  - % of local green space near home (amount of green space)  - Perceived access to local green space  - Perceived quality of local green space  - Nature relatedness  - Road coverage  - Environmental deprivation  - Visit frequency to local green space |
| Fox et al., 2011 [40]^‡^  England | Cross-sectional; quantitative non-randomised  UK National Prevention Research Initiative | 240 participants (age range: ≥ 70 years); 125 male, 115 female  SES (quartiles of IMD of participants’ residence):  Lower (3^rd^ and 4^th^ quartiles): 107 (115 for distance to nearest shop)  Higher (1^st^ and 2^nd^ quartiles): 107 (115 for distance to nearest shop) | Not stated | Measure: Device-based (ActiGraph GT1M accelerometer)  Outcome in review: Continuous |  |  |  |  |  |  |  |  |  | - Physical function (physical function)  - Body mass index (weight status)  - Distance to nearest shop (walking and cycling infrastructure)  - Weekly trips |
| Goodman et al., 2012 [41]^‡^  Wales, England  iConnect | Cross-sectional; quantitative non-randomised  Engineering and Physical Sciences Research Council  National Institute for Health Research  Medical Research Council  British Heart Foundation  Economic and Social Research Council  Wellcome Trust | 3,463 participants (age range: 18 to 96 years); 1,556 male, 1,900 female  SES (annual household income):  Lower (≤ £20,000): 876  Higher (> £20,000): 1,990  *Sub-sample:*  1,230 participants aged 60+ years  SES (annual household income):  Lower (≤ £20,000): 467 (body mass index), 477 (general health)  Higher (> £20,000): 494 (body mass index), 500 (general health) | Not stated | Outcome in review: Continuous | ? | ? | ? |  | - |  | ? | + | + | - Body mass index (weight status)  - General health (perception of general health) |
| Goodman et al., 2014 [42]^‡^  England, Wales  iConnect  Baseline: April 2010  Follow-up 1: April 2011  Follow-up 2: April 2012 | Prospective; quantitative non-randomised (2 years)  Engineering and Physical Sciences Research Council  Medical Research Council  British Heart Foundation  Economic and Social Research Council  National Institute for Health Research  Wellcome Trust  Baseline and follow-up 1 | 3,516 participants at baseline (age range: 18 to 89 years)  1,796 participants at follow-up 1; 817 male, 979 female  SES (annual household income):  Follow-up 1 sample:  Lower (≤ £20,000): 560  Higher (> £20,000): 1,109  *Sub-sample:*  782 participants aged 60+ years  SES (annual household income):  Lower (≤ £20,000): 391  Higher (> £20,000): 391 | Not stated | Measure: Adapted version of the short form of the International Physical Activity Questionnaire  Outcome in review: Continuous | ? | ? | - |  | + |  | - | + | + | - Exposure to the intervention, defined as proximity to Connect2, an initiative established with the intention of building or improving walking and cycling routes (walking and cycling infrastructure) |
| Goodwin et al., 2023 [43]^†^  England  English Longitudinal Study of Ageing  Wave 1, 2, 3, 4, 5, 6, 7, 8, 9 | Prospective; quantitative non-randomised (16 years)  Loughborough University  Wave 1, 2, 3 | 11,292 participants (mean age: 63.54 ± 9.77 years, no hearing loss; 67.29 ± 10.39 years, hearing loss); 2,540 male, 3,806 female (no hearing loss); 2,599 male, 2,347 female (hearing loss)  SES (wealth):  Lower (1^st^ to 3^rd^ quintiles): 3,492 (no hearing loss); 3,098 (hearing loss)  Higher (4^th^ and 5^th^ quintiles): 2,726 (no hearing loss); 1,779 (hearing loss)  *Sub-sample:*  7,167 participants aged 60+ years; 3,241 male, 3,926 female  SES (wealth):  Lower (1^st^ to 3^rd^ quintiles): 4,370  Higher (4^th^ to 5^th^ quintiles): 2,699 | Not stated | Outcome in review: Dichotomous | ? | ? | - | + | - |  | - | + | + | - Smoking, covariate (smoking status)  - Body mass index, covariate (weight status)  - Loneliness, covariate |
| Hanson et al., 2021 [88]^‡^  England  Active Northumberland | Cross-sectional; quantitative non-randomised  Not applicable | 20,904 participants (age range: 18 to 70+ years); 8,662 male, 12,237 female  SES (IMD quintiles):  Lower (1^st^ and 2^nd^ quintiles): 6,318  Higher (3^rd^ to 5^th^ quintiles): 13,743  *Sub-sample:*  5,178 participants aged 60+ years  SES (IMD quintiles):  Lower (1^st^ and 2^nd^ quintiles): 1,053  Higher (3^rd^ to 5^th^ quintiles): 3,943 | Not stated | Outcome in review: Dichotomous |  |  |  |  |  |  |  |  |  | - Leisure centre classification |
| Hillsdon et al., 2001 [44]^*^  England  ACTIVE for LIFE campaign prospective longitudinal survey  Wave 1, 2, 3 | Prospective; quantitative non-randomised (2 years)  Department of Health  Wave 1, 2 | 6,711 participants (age range: 16 to 74 years); 43.6% male  SES (social grade):  Lower (C2DE): 49.5%  Higher (ABC1): 50.4%  *Sub-sample:*  1,705 participants aged 60+ years; 728 male, 977 female  SES (social grade):  Lower (C2DE): 938  Higher (ABC1): 764 | Not stated | Outcome in review: Dichotomous | ? | ? | ? | + | + |  | ? | + | + | - Readiness to change (motivation)  - Knowledge of the moderate intensity recommendation  - Barriers to physical activity |
| Houdmont et al., 2015 [45]^*^  Northern Ireland  Stormont Study | Cross-sectional; quantitative non-randomised  Doughty Fund of the Faculty of Occupational Medicine, Royal College of Physicians of Ireland | 3,795 participants (age range: 21 to 70 years); 1,727 male, 2,068 female  *Sub-sample:*  103 participants aged 60+ years; 49 male, 54 female  SES (salary band):  Lower (< £20,000): 15  Higher (≥ £20,000): 88 | Not stated | Outcome in review: Dichotomous | ? | ? | ? | + | + |  | ? | + | + | - Body mass index (weight status)  Psychosocial work environment:  - Demands  - Control  - Managerial support  - Peer support  - Relationships  - Role  - Change |
| Hudson et al., 2015 [46]^‡^  Northern Ireland  Northern Ireland Health Survey  Wave 1 | Cross-sectional; quantitative non-randomised  CARDI data-mining call 2013  Wave 1 | 1,975 participants from Northern Ireland (age range: 50 to 80+ years); 46% male  *Sub-sample:*  1,325 participants aged 60+ years  SES (gross household income quartiles):  Lower (1^st^ and 2^nd^ quartiles): 675  Higher (3^rd^ and 4^th^ quartiles): 650 | Not stated | Measure: International Physical Activity Questionnaire  Outcome in review: Dichotomous | + | ? | + | + | + |  | - | + | + | - Self-rated health (perception of general health) |
| Jackson and Steptoe, 2017 [89]^†^  England  English Longitudinal Study of Ageing  Wave 4, 5 | Cross-sectional; quantitative non-randomised  Cancer Research UK  Wave 4, 5 | 5,480 participants (mean age: 67.71 ± 8.95 years, no weight discrimination; 62.54 ± 6.89 years, weight discrimination; age range: ≥ 50 years); 2,423 male, 3,057 female  SES (household non-pension wealth quintiles):  Lower (1^st^ and 2^nd^ quintiles): 1,937  Higher (3^rd^ to 5^th^ quintiles): 3,543  *Sub-sample:*  4,343 participants aged 60+ years; 1,928 male, 2,415 female  SES (household non-pension wealth quintiles):  Lower (1^st^ and 2^nd^ quintiles): 1,481  Higher (3^rd^ to 5^th^ quintiles): 2,862 | Not stated | Outcome in review: Dichotomous | ? | ? | - | + | - |  | - | + | + | - Perceived weight discrimination |
| Jackson et al., 2019 [90]^†^  England  English Longitudinal Study of Ageing  Wave 6 | Cross-sectional; quantitative non-randomised  Cancer Research UK  Wave 6 | 5,861 participants (mean age: 70.88 ± 7.36 years, does not have a concessionary bus pass; mean age: 71.83 ± 6.87 years, has a concessionary bus pass; age range: ≥ 62 years); 2,612 male, 3,249 female  SES (household non-pension wealth quintiles):  Lower (1^st^ and 2^nd^ quintiles): 1,979  Higher (3^rd^ to 5^th^ quintiles): 3,882 | Not stated | Outcome in review: Dichotomous | ? | ? | - | + | - |  | - | + | + | - Bus pass ownership  - Frequency of bus pass use |
| Johnson et al., 2011 [47]^‡^  Scotland  Lothian Birth Cohort 1936  Baseline: 1947  Follow-up: recruited from 2004 to 2007 | Prospective; quantitative non-randomised (57–60 years)  Research Councils UK  Medical Research Council  University of Edinburgh  Biotechnology and Biological Sciences Research Council  Engineering and Physical Sciences Research Council  Follow-up | 1,091 participants (mean age: 69.6 ± 0.8 years, age range: 67.7 to 71.3 years); 548 male, 543 female  SES (SIMD at age 70):  Lower (strictly below the median): 456  Higher (above or equal to the median): 498 | Not stated | Outcome in review: Continuous | ? | ? | ? | + | - |  | ? | + | + | - Age-70 IQ-scaled Moray House Test  - Body mass index (weight status)  - HADS Anxiety  - HADS Depression (depressive symptoms)  - Pack-years of smoking (smoking status)  - Weekly units of alcohol consumed (alcohol consumption) |
| Kobayashi et al., 2016 [48]^†^  England  English Longitudinal Study of Ageing  Wave 2, 3, 4, 5, 6 | Prospective; quantitative non-randomised (8 years)  Canadian Institutes of Health Research  University College London  Cancer Research UK  Wave 2, 3, 4, 5, 6 | 4,345 participants (mean age: 63.28 ± 7.26 years; age range: 52 to 79 years); 1,913 male, 2,432 female  SES (net non-pension wealth quintiles):  Lower (1^st^ and 2^nd^ quintiles): 1,392  Higher (3^rd^ to 5^th^ quintiles): 2,738  2,697 community-dwelling participants aged 60+ years; 1,168 male, 1,529 female  SES (net non-pension wealth quintiles):  Lower (1^st^ and 2^nd^ quintiles): 1,057  Higher (3^rd^ to 5^th^ quintiles): 1,606 | Not stated | Outcome in review: Dichotomous | ? | ? | - | + | - |  | - | + | + | - Health literacy (health literacy)  - Memory (memory)  - Verbal fluency |
| Koetsenruijter et al., 2015 [49]^*^  UK  EU-WISE project | Cross-sectional; quantitative non-randomised  European Union’s Seventh Framework Programme for research, technological development and demonstration | 1,692 participants (mean age: 66.2 years); 50.0% female  SES (income relative to the country’s average income):  Lower (below the country yearly average income): 61.0%  Higher (about or above the country yearly average income): 39.0%  *Sub-sample:*  Of 288 (281 in publication) UK participants in dataset, 213 aged 60+ years; 126 male, 75 female, 12 missing information on biological sex  SES (income relative to the country’s average income):  Lower (below the country yearly average income): 138  Higher (about or above the country yearly average income): 67 | Not stated | Measure: Rapid Assessment of Physical Activity  Outcome in review: Dichotomous | ? | ? | ? | + | + |  | ? | + | + | - Support network members (social support)  - Network members providing information support  - Network members providing practical support  - Network members providing emotional support  - Health professional in wider network  - Attending community organizations (social participation) |
| Lamb, 1992 [91]^*^  England | Cross-sectional; quantitative non-randomised  Not reported | 118 participants (mean age: 37.90 ± 11.72; age range: 18 to 64 years); 77 male, 41 female  *Sub-sample:*  3 participants aged 60+ years; 3 male, 0 female  SES (occupational class):  Lower (manual): 1  Higher (non-manual): 2 | Not stated | Measure: Liverpool Leisure-Time Physical Activity Questionnaire  Outcome in review: Dichotomous | + | ? | + |  | + |  | ? | + | + | - Self-perceived fitness I  - Self-perceived fitness II |
| Lemanska et al., 2019 [50]^‡^  England  Baseline  3-month follow-up  6-month follow-up | Prospective; quantitative non-randomised (6 months)  Movember Foundation  Prostate Cancer UK  National Institute for Health and Care Research Clinical Research Network  Baseline and 3-month follow-up | 116 participants (mean age: 70.4 ± 7.2 years; age range: 50 to 85 years); 116 male, 0 female  SES (IMD deciles):  Lower (deciles 1–3): 16  Higher (deciles 4–10): 100  *Sub-sample:*  106 participants aged 60+ years; 106 male, 0 female  SES (IMD deciles):  Lower (deciles 1–3): 15  Higher (deciles 4–10): 91 | Medical Research Council’s complex intervention framework | Measure: Device-based (ActiWatch V.4.0 accelerometer)  Outcome in review: Dichotomous |  |  |  |  |  |  |  |  |  | - Body mass index (weight status) |
| Luciano et al., 2010 [51]^‡^  Scotland  Generation Scotland’s Scottish Family Health Study | Cross-sectional; quantitative non-randomised  Chief Scientist Office of the Scottish Executive Health Department  Biotechnology and Biological Sciences Research Council  Engineering and Physical Sciences Research Council  Economic and Social Research Council  Medical Research Council  Royal Society of Edinburgh  Lloyds TSB Foundation for Scotland  Chief Scientist Office at the Scottish Government Health Directorates  Wellcome Trust | 6,118 participants (mean age: 47.4 ± 14.5 years; age range: 18 to 99 years); 3,639 female  SES (average income), 5,185 participants:  Lower (up to 30 k): 10.7%  Higher (above 30 k): 78.6%  No answer: 10.7%  *Sub-sample:*  1,319 participants aged 60+ years  SES (average income):  Lower (up to 30 k): 664 (604 with physical activity measured)  Higher (above 30 k): 345 (333 with physical activity measured) | Not stated | Outcome in review: Dichotomous | ? | ? | ? | + | - |  | ? | + | + | - Digital symbol  - Verbal fluency  - Mill Hill  - Logical memory (memory)  - General cognitive ability factor |
| Macdonald, 2019 [52]^*^  Scotland  Transport, Housing and Well-being Study  2010 | Cross-sectional; quantitative non-randomised  UK Medical Research Council Neighbourhoods and Communities Programme  Chief Scientist Office  2010 | 513 participants (mean age: 44.9 years; age range: 19 to 74 years); 220 male, 293 female  SES (income deprivation tertiles):  Lower (highest tertile): 180  Higher (lowest or middling tertiles): 333  *Sub-sample:*  47 participants aged 60+ years; 33 male, 14 female  SES (income deprivation tertiles):  Lower (highest tertile): 15  Higher (lowest or middling tertiles): 32 | Not stated | Outcome in review: Continuous | ? | ? | ? |  | + |  | ? | + | + | - Public or private physical activity facility access, within 800 m of home or workplace neighbourhood (built physical activity facilities)  - Physical activity facility proximity  - Physical activity facility location  - Physical activity facility type |
| Mackenbach et al., 2016 [53]^‡^  England  Sustainable prevention of obesity through integrated strategies (SPOTLIGHT) project | Cross-sectional; quantitative non-randomised  Seventh Framework Programme of the European Commission, HEALTH | 6,037 participants (age range: ≥ 18 years); 5,205 participants for analyses  *Sub-sample:*  526 UK participants, of which 173 aged 60+ years  SES (educational level):  Lower (from less than primary to higher secondary education): 79  Higher (college or University level): 66 | Socio-ecological models of health behaviour | Measure: International Physical Activity Questionnaire  Outcome in review: Dichotomous | + | ? | + | + | + |  | - | + | + | - Presence of parks (natural physical activity facilities)  - Presence of indoor recreational facilities (built physical activity facilities)  - Presence of outdoor recreational facilities  - Presence of bicycle lanes (walking and cycling infrastructure)  - Number of individual perceived physical activity barriers |
| Marques et al., 2015 [54]^¶^  UK  European Social Survey  Round 6 (2012) | Cross-sectional; quantitative non-randomised  Not reported  Round 6 (2012) | 52,936 participants (mean age: 49.4 ± 18.0 years; age range: 18 to 65+ years); 24,096 male, 28,840 female  SES (education level):  Lower (less than high school/high school): 40,869  Higher (superior education): 12,067  *Sub-sample:*  Of 2,441 UK participants, 865 aged 60+ years; 379 male, 486 female  SES (education level):  Lower (less than high school/high school): 627  Higher (superior education): 211 | Not stated | Outcome in review: Dichotomous | ? | ? | ? | + | + |  | ? | + | + | - Perception of health (perception of general health) |
| Morrison et al., 2004 [92]^∮^  Scotland  1^st^ survey: June 27, 2000  2^nd^ survey: June 28, 2001 | Prospective; quantitative non-randomised (1 year)  Chief Scientist Office of the Scottish Executive Department of Health  1^st^ and 2^nd^ survey | 244 participants, 1^st^ survey (age range: 15 to 75+ years); 82 male, 160 female; 185 participants, 2^nd^ survey (age range: 15 to 75+ years); 63 male, 120 female  SES (deprived urban housing estate):  Lower: 100%  Higher: 0% | Not stated | Outcome in review: Continuous |  |  |  |  |  |  |  |  |  | - Introduction of the traffic calming scheme |
| Ogilvie et al., 2008 [55]^‡^  Scotland | Cross-sectional; quantitative non-randomised  Medical Research Council  Chief Scientist Office of the Scottish Executive Health Department | 1,322 participants (median age: 48 years; age range: 16 to 89 years); 804 female  SES (deprived urban population):  Lower: 100%  Higher: 0%  *Sub-sample:*  164 participants aged 60+ years (physical activity); 201 participants aged 60+ years (active travel)  SES (deprived urban population):  Lower: 100%  Higher: 0% | Not stated | Measure: Short form of the International Physical Activity Questionnaire  Outcome in review: Dichotomous | + | ? | + | + | + |  | - | + | + | - Perceived proximity to shops (walking and cycling infrastructure)  - Perceived road safety for cyclists  - Perception of traffic volume  - Body mass index (weight status)  - Difficulty walking for a quarter of a mile (physical function) |
| Oliveira et al., 2022 [56]^‡^  UK | Cross-sectional; quantitative non-randomised  Alzheimer's Research UK Midlands Network Centre | 3,948 participants (mean age: 62.3 ± 7.9 years); 1,060 male, 2,880 female  SES (highest qualification):  Lower (non-graduate): 1,651  Higher (graduate or post-graduate): 2,297  *Sub-sample:*  2,326 participants aged 60+ years  SES (highest qualification):  Lower (non-graduate): 1,003  Higher (graduate or post-graduate): 1,323 | Not stated | Outcome in review: Dichotomous | ? | ? | ? | + | - |  | ? | + | + | - General health (perception of general health)  - Consumes alcohol in a typical month (alcohol consumption)  - Smoker (smoking status)  - Feel low or depressed (depressive symptoms)  - Feel stressed  - Social activity (social participation)  - Mental activity (memory)  - Sleep well (sleep) |
| Pina et al., 2021 [57]^‡^  Scotland | Cross-sectional; quantitative non-randomised  Medical Research Council Confidence in Global Nutrition and Health Research  University of Stirling | 150 participants in the Scottish cohort (median age, interquartile range: 69, 66–73 years; age range of inclusion criteria: 60 to 85 years); 117 female  *Sub-sample:*  SES (education), Scottish cohort:  Lower (less than secondary/completed secondary): 40 (38 in analyses)  Higher (completed tertiary): 110 (112 in analyses) | Not stated | Measure: Device-based (ActiGraph GT3X+ accelerometer)  Outcome in review: Continuous |  |  |  |  |  |  |  |  |  | - Body mass index, covariate (weight status)  - Smoking status, covariate (smoking status) |
| Pliakas et al., 2014 [93]^∮^  England  Whitehall II study  Phase 7 | Cross-sectional; quantitative non-randomised  Phase 7  UK Economic and Social Research Council  Greek State Scholarship Foundation | 3,363 participants (mean age: 61 years; age range: 50 to 74 years); 65.3% male  SES (current/most recent employment grade at the civil service):  Low (executive officer, clerical/support): 14.6%  Medium (senior or higher executive officer)  High (unified grade 1–6, 7) | Social-ecological models of behaviour | Measure: Adapted version of the Minnesota Leisure-Time Physical Activity Questionnaire  Outcome in review: Continuous | + | ? | + |  | + |  | - | + | + | - Crime score  - Density of non-residential buildings  - Density of road network  - Density of public green space  - Population weighted road km to a food store  - Annual average NO_2_ concentration  - Killed and seriously injured per km of road  - Access to public transport |
| Poortinga, 2007 [58]^†^  England  Health Survey for England  2003 | Cross-sectional; quantitative non-randomised  Not reported  2003 | 11,617 participants (age range: 16 to 64 years); 45.1% male, 54.9% female  SES (household social class):  Lower (manual): 41.5%  Higher (non-manual): 55.8%  *Sub-sample:*  1,050 participants aged 60+ years; 489 male, 561 female  SES (household social class):  Lower (manual): 508  Higher (non-manual): 527 | Not stated | Outcome in review: Dichotomous | ? | ? | ? | + | + |  | ? | + | + | - Sportsclub membership (social participation) |
| Renton et al., 2012 [59]^∮^  England  Well London survey  Baseline | Cross-sectional; quantitative non-randomised  Wellcome Trust  Baseline | 4,107 participants (age range: 16 to 65+ years); 2,077 participants for complete case analysis  SES (deprived census lower super output areas based on the English IMD):  Lower: 100%  Higher: 0% | Not stated | Measure: International Physical Activity Questionnaire  Outcome in review: Dichotomous | + | ? | + | + | + |  | - | + | + | - Creative activity participation (social participation) |
| Rogers et al., 2020 [60]^*^  UK | Cross-sectional; quantitative non-randomised  Not reported | 9,190 participants (age range: 20 to 70+ years); 1,914 male, 7,143 female, 73 all other genders  SES (household income):  Lower (< £25,000): 2,542  Higher (£25,000 and above): 5,434  *Sub-sample:*  1,062 participants aged 70+ years; 290 male, 754 female, 4 all other genders  SES (household income):  Lower (< £25,000): 398  Higher (£25,000 and above): 458 | Not stated | Outcome in review: Dichotomous | ? | ? | ? | + | - |  | ? | + | + | - Disability, difficulties in activities of daily living (physical function)  - Depression (depressive symptoms)  - Obesity (weight status) |
| Rowlands et al., 2021 [61]^‡^  England  Chronotype of Patients with Type 2 Diabetes and Effect on Glycaemic Control  Baseline: 2017–2020  Follow-up: May 17 to June 12, 2020 | Prospective; quantitative non-randomised (up to 3 years; median, interquartile range: 438, 253–760 days)  National Institute for Health Research Leicester Biomedical Research Centre, a partnership between University Hospitals of Leicester National Health Service Trust, Loughborough University and the University of Leicester  Baseline and follow-up | 885 participants (age range: 18 to 75 years); 165 with accelerometer data both before and during COVID (mean age: 64.2 ± 8.3 years); 74 female  *Sub-sample:*  114 participants aged 60+ years  SES (IMD rank):  Lower (strictly below the median): 56  Higher (above or equal to the median): 58 | Not stated | Measure: Device-based (GENEActiv accelerometer)  Outcome in review: Continuous |  |  |  |  |  |  |  |  |  | - Body mass index (weight status)  - Depressive symptoms (depressive symptoms)  - Alcohol intake (alcohol consumption)  - Smoking status (smoking status)  - Short Physical Performance Battery (physical function)  - Haemoglobin A1c  - Sleep duration (sleep) |
| Rütten and Abu-Omar, 2004 [94]^†^  Great Britain (Scotland, England, Wales)  Eurobarometer 58.2  2002 | Cross-sectional; quantitative non-randomised  Not reported  2002 | 16,230 participants (age range: 15 to 65+ years); 7,500 male, 8,730 female  SES (gross household income: quartile of national income):  Lower (two lowest quartiles): 5,550  Higher (two highest quartiles): 5,172  *Sub-sample:*  Of 1,010 participants from Great Britain, 188 aged 60+ years; 74 male, 114 female  SES (gross household income: quartile of national income):  Lower (two lowest quartiles): 77  Higher (two highest quartiles): 29 | Not stated | Measure: Short form of the International Physical Activity Questionnaire  Outcome in review: Dichotomous | ? | ? | ? | + | + |  | ? | + | + | - “The area where I live offers me many opportunities to be physically active”  - “Local sport clubs and other providers offer many opportunities to be physically active”  - “My local authority does enough for its citizens concerning their physical activities” |
| Salman et al., 2019 [62]^†^  Scotland  Scottish Health Survey  2014–2017 | Cross-sectional; quantitative non-randomised  Not applicable  2014–2017 | 1,259 participants (age range: ≥ 16 years); 669 male, 586 female  SES (SIMD quintiles):  Lower (1^st^ and 2^nd^ quintiles):  558  Higher (3^rd^ to 5^th^ quintiles): 697  *Sub-sample:*  883 participants aged 60+ years; 469 male, 414 female  SES (SIMD quintiles):  Lower (1^st^ and 2^nd^ quintiles): 373  Higher (3^rd^ to 5^th^ quintiles): 510 | Not stated | Measure: Questions based on the Allied Dunbar National Fitness Survey  Outcome in review: Dichotomous | ? | ? | ? | + | + |  | ? | + | + | - Smoking status (smoking status)  - Body mass index (weight status)  - Portion of fruit and vegetables (fruit and vegetable consumption)  - Mental wellbeing (depressive symptoms) |
| Scholes and Mindell, 2020 [63]^‡^  England  Health Survey for England 2008, 2012, 2016 | Cross-sectional; quantitative non-randomised  2008, 2012, 2016 | 24,882 participants (age range: ≥ 16 years); 11,199 male, 13,683 female  SES (equivalised household income tertiles):  Lower (lowest tertile): 7,802  Higher (middle/highest tertiles): 17,080  *Sub-sample:*  7,724 participants aged 60+ years; 3,622 male, 4,102 female  SES (equivalised household income tertiles):  Lower (lowest tertile): 3,056  Higher (middle/highest tertiles): 4,668 | Not stated | Measure: Physical Activity and Sedentary Behaviour Assessment Questionnaire  Outcome in review: Continuous | + | ? | + | + | + |  | - | + | + | - Body mass index, covariate (weight status)  - Smoking status, covariate (smoking status)  - Self-rated health, covariate (perception of general health) |
| Sheeran et al., 2013 [64]^‡^  England  Baseline  Follow-up 1: 1 month  Follow-up 2: 7 months | Prospective; quantitative randomised controlled trials (7 months)  Not reported  Baseline and follow-up 2 | 467 participants (mean age: 53.88 ± 12.42 years); 467 male, 0 female  SES (occupational class):  Lower (predominantly working class, with unskilled or semiskilled jobs): 100%  *Sub-sample:*  28 participants aged 60+ years; 28 male, 0 female  SES (occupational class):  Lower (predominantly working class, with unskilled or semiskilled jobs): 100% | - Fantasy realisation theory  - Theory of Planned Behaviour | Measure: Godin-Shephard Leisure-Time Physical Activity Questionnaire  Outcome in review: Continuous | + | ? | - | - | + | ? | - | + | + | - Instrumental attitude  - Intention (motivation)  - Perceived behavioural control  - Subjective norm  - Affective attitude |
| Smith et al., 2019 [65]^‡^  UK  UK Biobank  Baseline data (2006–2010) and device-measured physical activity data (2013–2015) | Cross-sectional; quantitative non-randomised  Medical Research Council  British Heart Foundation  Economic and Social Research Council  National Institute for Health Research  Wellcome Trust  2006–2010 and 2013–2015 | 502,633 participants (age range: 37 to 73 years at recruitment); 229,171 male, 273,462 female  65,967 participants provided recorded physical activity data (age range: 40 to 79 years); 28,718 male, 37,249 female  *Sub-sample:*  41,662 participants aged 60+ years provided recorded physical activity data  SES (Townsend deprivation index quintiles):  Lower (4^th^ and 5^th^ quintiles): 13,563  Higher (1^st^ to 3^rd^ quintiles): 28,099 | Not stated | Measure: Device-based (Axivity AX3 accelerometer)  Outcome in review: Dichotomous |  |  |  |  |  |  |  |  |  | - Facilities for physical activity (built physical activity facilities)  - Parks (natural physical activity facilities)  - Walkability (walking and cycling infrastructure)  - Air pollution  - Noise pollution  - Distance to major road  - Terrain  - Greenness (amount of green space) |
| Sniehotta et al., 2013 [95]^∮^  Scotland  Physical Activity Cohort Scotland  Wave 1 | Cross-sectional; quantitative non-randomised  Scottish Executive (funder)  University of Dundee (sponsor)  Fuse, the Centre for Translational Research in Public Health  British Heart Foundation  Cancer Research UK  Economic and Social Research Council  Medical Research Council  National Institute for Health Research  Wave 1 | 584 participants (mean age: 78.4 ± 7.7 years; age range: 65 to 105 years); 45.7% male, 54.3% female  SES (SIMD score deciles) | - Social cognitive theories  - Theory of planned behaviour | Measure: Device-based (RT3 accelerometer)  Outcome in review: Continuous |  |  |  |  |  |  |  |  |  | - Intention  - Self-efficacy |
| Solomon et al., 2013 [66]^‡^  England | Cross-sectional; quantitative non-randomised  Economic and Social Research Council  National Institute for Health Research Collaborations for Leadership in Applied Health Research and Care | 2,415 participants (mean age: 58.0 ± 15.2 years; age range: 18 to 102 years); 37.3% male, 62.7% female  *Sub-sample:*  1,164 participants aged 60+ years  SES (IMD):  Lower (more deprived than the median score for villages in Devon): 538  Higher (less deprived than or equal to the median score for villages in Devon): 626 | Not stated | Measure: Short form of the International Physical Activity Questionnaire  Outcome in review: Continuous | + | ? | + | + | + |  | - | + | + | - Dog ownership (dog ownership)  - Health status (perception of general health)  - Body mass index (weight status)  - Commitment to doing more physical activity (motivation)  - Physical activity social norms  - Physical activity habit  - Physical activity village supportiveness  - Traffic and pleasantness of surroundings  - Proximity and convenience of walking (walking and cycling infrastructure)  - Safety and convenience of cycling  - Convenience of public transport  - Safety of walking after dark  - Manmade sports facilities in local area (built physical activity facilities)  - Natural activity facilities in the local area (natural physical activity facilities)  - Community centre/village hall in local area  - Use of recreational facilities  - Locality of facilities used |
| Stephan et al., 2022 [96]^‡^  England  English Longitudinal Study of Ageing  Wave 5, 6 | Prospective; quantitative non-randomised (2 years for the English Longitudinal Study of Ageing sample)  National Institute on Aging of the National Institutes of Health  Wave 5 | 5,988 English Longitudinal Study of Ageing participants (mean age: 66.21 ± 8.26 years; age range: 50 to 89 years); 55% female  *Sub-sample:*  3,852 English Longitudinal Study of Ageing participants aged 60+ years  SES (education):  Lower (below degree): 3,022  Higher (degree or equivalent): 830 (780 for neuroticism) | Five-Factor Model | Outcome in review: Continuous | ? | ? | - |  | - | ? | - | + | + | - Neuroticism  - Extraversion  - Openness  - Agreeableness  - Conscientiousness |
| Steptoe and Fancourt, 2020 [97]^†^  England  English Longitudinal Study of Ageing  Wave 6, 7, 8 | Prospective; quantitative non-randomised (4 years)  Wellcome Trust  Wave 6, 7, 8 | 5,694 participants (mean age: 66.65 ± 8.68 years; age range: 52 to 90+ years); 2,529 male, 3,165 female  SES (education):  Lower (low/intermediate): 3,713  Higher (higher): 1,981  *Sub-sample:*  4,375 community-dwelling participants aged 60+ years; 1,972 male, 2,403 female  SES (education):  Lower (low/intermediate): 2,915  Higher (higher): 1,460 | Not stated | Outcome in review: Dichotomous | ? | ? | - | + | - |  | - | + | + | - Worthwhile ratings |
| Stevinson et al., 2014 [67]^‡^  UK | Cross-sectional; quantitative non-randomised  Macmillan Cancer Support | 748 participants (mean age: 65.4 ± 10.2 years); 229 male, 510 female  SES (IMD):  Lower (most deprived): 233  Higher (least deprived): 372  *Sub-sample:*  567 participants aged 60+ years  SES (IMD):  Lower (most deprived): 166  Higher (least deprived): 293 | Not stated | Measure: Leisure Time Exercise Questionnaire  Outcome in review: Continuous | + | ? | - | + | + |  | - | + | + | - Health status (perception of general health) |
| Strain et al., 2022 [68]^‡^  England  Active Lives Survey  2015–2019 and 2020 | Repeated cross-sectional; quantitative non-randomised  UK Medical Research Council  National Institute for Health and Care Research Biomedical Research Centre in Cambridge  2020 | 69,889 participants, weighted sample size (age range: 16 to 85+ years); 34,296 male, 35,500 female, 93 other  SES (IMD deciles):  Lower (1^st^ to 5^th^ deciles): 37,971  Higher (6^th^ to 10^th^ deciles): 31,918  *Sub-sample:*  6,246 participants aged 65+ years in 2020  SES (IMD deciles):  Lower (1^st^ to 5^th^ deciles): 3,490  Higher (6^th^ to 10^th^ deciles): 2,756 | Not stated | Outcome in review: Dichotomous and continuous | ? | ? | ? | + | + |  | ? | + | + | - Body mass index, covariate (weight status) |
| Thogersen-Ntoumani et al., 2017 [98]^‡^  UK (any/all) | Cross-sectional; quantitative non-randomised  Not reported | 217 participants (age range: 50 to 77 years; mean age: 57.1 ± 5.6 years); 69.1% male  SES (occupational class):  Lower (manual): 217  Higher (non-manual): 0  *Sub-sample:*  63 participants aged 60+ years  SES (occupational class):  Lower (manual): 63  Higher (non-manual): 0 | Not stated | Measure: Baecke Questionnaire  Outcome in review: Continuous | + | ? | + | - | + |  | - | + | + | - Time demands  - Physical demands  - Mental demands  - Interpersonal demands  - Output demands  - Stress resilience  - High pleasure/low arousal  - High pleasure/high arousal  - Low pleasure/low arousal  - Low pleasure/high arousal  - Self-rated health |
| Timmermans et al., 2017 [69]^‡^  England  Sustainable prevention of obesity through integrated strategies (SPOTLIGHT) project | Cross-sectional; quantitative non-randomised  Seventh Framework Programme of the European Commission, HEALTH | 6,037 participants; 5,900 included in analyses (age range: 18 to 109 years; mean age: 52 years); 13.4% UK; 44% male  SES (level of education):  Lower (lower): 69.7%  *Sub-sample:*  236 UK participants aged 60+ years  SES (level of education):  Lower (lower): 137  Higher (either a college or University degree): 99 | Conceptual framework of Patel and Hu | Measure: International Physical Activity Questionnaire  Outcome in review: Dichotomous | + | ? | + | + | + |  | - | + | + | - Sleep duration (sleep) |
| Vijaykumar et al., 2021 [70]^†^  UK (any/all) | Cross-sectional; quantitative non-randomised  Consumer Data Research Centre, an Economic Social and Research Council Data Investment | 676 participants (age range: 18 to 75 years); 335 male, 341 female  SES (household income):  Lower (below £30,000): 396  Higher (£30,000 and above): 280  *Sub-sample:*  109 participants aged 65+ years; 89 male, 20 female  SES (household income):  Lower (below £30,000): 61  Higher (£30,000 and above): 48 | Model of the influence of different media sources on nutrition-related confusion and backlash, and subsequent health behaviours | Outcome in review: Dichotomous | ? | ? | ? | + | - |  | ? | + | + | - Nutrition confusion  - Nutrition backlash  - Health status (perception of general health)  - Health consciousness  - Trust in traditional and online media  - Servings of fruit and vegetables (fruit and vegetable consumption) |
| von Wagner et al., 2007 [71]^*^  All of the UK except for the Scottish Isles and Northern Ireland | Cross-sectional; quantitative non-randomised  Cancer Research UK  British Heart Foundation | 719 participants (mean age: 47.2 ± 18.3 years); 304 male, 415 female  SES (annual personal income):  Lower (up to £19,999): 437  Higher (more than £20,000): 172  *Sub-sample:*  204 participants aged 60+ years; 98 male, 106 female  SES (annual personal income):  Lower (up to £19,999): 130  Higher (more than £20,000): 26 | Not stated | Outcome in review: Dichotomous | ? | ? | ? | + | - |  | ? | + | + | - Functional health literacy (health literacy) |
| Wang et al., 2022 [99]^‡^  UK (any/all)  Platform for Research Online to Investigate Genetics and Cognition in Aging (PROTECT)  May and June 2020 | Cross-sectional; quantitative non-randomised  Not applicable  May and June 2020 | 3,807 participants (mean age: 66.77 ± 6.83 years); 79.64% female  SES (education):  Lower (secondary/post-secondary): 23.33%  Higher (vocational/undergraduate/post-graduate/doctorate): 76.68%  *Sub-sample:*  3,203 participants aged 60+ years  SES (education):  Lower (secondary/post-secondary): 772  Higher (vocational/undergraduate/post-graduate/doctorate): 2,431 | Not stated | Outcome in review: Dichotomous | ? | ? | ? | + | - |  | ? | + | + | - Familiarity with social media |
| Wardle and Griffith, 2001 [72]^†^  Great Britain (England, Scotland, Wales)  Omnibus survey of the Office of National Statistics  March 1999 | Cross-sectional; quantitative non-randomised  Economic and Social Research Council  March 1999 | 1,790 participants (mean age, men: 46.1 years; mean age, women: 47.2 years); 887 male, 903 female  SES (social class):  Lower (manual): 739  Higher (non-manual): 1,051  *Sub-sample:*  555 participants aged 60+ years; 235 male, 320 female  SES (social class):  Lower (manual): 247  Higher (non-manual): 281 | Not stated | Outcome in review: Dichotomous | ? | ? | ? | + | + |  | ? | + | + | - Body mass index, covariate (weight status) |
| Wardle and Steptoe, 2003 [73]^†^  Great Britain (England, Scotland, Wales)  Omnibus survey of the Office of National Statistics  September 2000 | Cross-sectional; quantitative non-randomised  Cancer Research UK  September 2000 | 1,691 participants (age range: ≥ 16 years); 758 male, 933 female  SES (social class):  Lower (manual): 319  Higher (non-manual): 1,254  *Sub-sample:*  550 participants aged 60+ years; 242 male, 308 female  SES (social class):  Lower (manual): 132  Higher (non-manual): 384 | Not stated | Outcome in review: Dichotomous | ? | ? | ? | + | - |  | ? | + | + | - Self-rated health (perception of general health)  - Chance locus of control  - Future salience  - Expectations of longevity  - Health consciousness |
| Watts et al., 2017 [74]^†^  England  English Longitudinal Study of Ageing  Wave 1, 2, 3, 4, 5, 6, 7 | Prospective; quantitative non-randomised (12 years)  Not reported  Wave 1, 2 | 11,345 participants (age range: ≥ 50 years); 5,146 male, 6,198 female  SES (highest education qualification):  Lower (no qualifications): 5,353  Higher (intermediate qualifications/higher education or above): 4,999  *Sub-sample:*  5,402 participants aged 60+ years with data available at Wave 2; 2,400 male, 3,002 female  SES (highest education qualification):  Lower (NVQ2 or below): 3,572  Higher (NVQ3 or above): 1,317 | Frailty by accumulated deficits | Outcome in review: Dichotomous | ? | ? | - | + | - |  | - | + | + | - Sports club membership (social participation) |
| Webb et al., 2016 [100]^†^  England  English Longitudinal Study of Ageing  Wave 6 | Cross-sectional; quantitative non-randomised  Department of Health Policy Research Programme  National Institute for Health Research  Wave 6 | 4,650 participants (age range: ≥ 62 years); 2,091 male, 2,559 female  SES (nonpension household wealth quintiles):  Lower (1^st^ and 2^nd^ quintiles): 1,417  Higher (3^rd^ to 5^th^ quintiles): 3,233 | Not stated | Outcome in review: Dichotomous | ? | ? | - | + | - |  | - | + | + | - Difficulties with activities of daily living, covariate  - Mobility problems, covariate  - Frequency of use of public transport, covariate |
| Westgarth et al., 2019 [75]^*^  England | Cross-sectional; quantitative non-randomised  UK Medical Research Council  National Health and Medical Research Council  National Heart Foundation  Royal Canin | 646 adult non-dog owners and dog owners (age range: 13 to 99 years); 289 male, 347 female  SES (household gross income):  Lower (£0-£20,000): 141  Higher (£20,000 and above): 379  *Sub-sample:*  154 participants aged 70+ years; 78 male, 76 female  SES (household gross income):  Lower (£0-£20,000): 54  Higher (£20,000 and above): 61 | Not stated | Measure: Adapted from the RESIDential Environment Neighbourhood Physical Activity Questionnaire and Dogs and Physical Activity Tool  Outcome in review: Dichotomous and continuous | + | ? | + | + | + |  | ? | + | + | - Dog ownership (dog ownership)  - Family social support for walking, covariate (social support)  - Friend social support for walking, covariate  - Extraversion, covariate  - Agreeableness, covariate  - Conscientiousness, covariate  - Emotional stability, covariate  - Open to experiences, covariate  - Weight status, covariate (weight status)  - Self-rated general health, covariate (perception of general health) |
| Wielgoszewska et al., 2022 [76]^¶^  England  English Longitudinal Study of Ageing  Wave 9, Wave 1 of COVID-19 sub-study | Prospective; quantitative non-randomised (up to 2 years for the English Longitudinal Study of Ageing sample)  National Core Studies  UK Research and Innovation  National Institute for Health and Care Research  Health and Safety Executive  Medical Research Council  Wave 9, Wave 1 of COVID-19 sub-study | 2,417 English Longitudinal Study of Ageing participants (age range: 52 to 66 years); 48.0% male, 52.0% female  SES (education):  Lower (less than degree): 75.0%  Higher (degree): 25.0%  *Sub-sample:*  Of 2,417 English Longitudinal Study of Ageing participants, 1,320 aged 60+ years; 550 male, 770 female  SES (education):  Lower (less than degree): 925  Higher (degree): 395 | Not stated | Outcome in review: Dichotomous | ? | ? | ? | + | - |  | ? | + | + | - Psychological distress, covariate (depressive symptoms)  - Self-rated health, covariate (perception of general health) |
| Withall et al., 2011 [101]^∮^  England | Cross-sectional; mixed-methods study  British Heart Foundation  National Institute for Health Research | 152 participants, quantitative sample (age range: < 18 years to 55+ years); 32 male, 112 female  SES (deprived area):  Lower: 152  Higher: 0  *Sub-sample:*  82 participants aged 55+ years  SES (deprived area):  Lower: 82  Higher: 0 | Not grounded in an individual theory of behaviour | Not applicable |  |  |  |  |  |  |  |  |  | Motivations for exercise:  - Interest/Enjoyment  - Social  - Appearance  - Competence  - Fitness |
| Wyke et al., 2019 [77]^‡^  England  Baseline  Follow-up 1: post-programme  Follow-up 2: 12 months | Prospective; quantitative randomised controlled trials (1 year)  European Union’s Seventh Framework Program for research, technological development, and demonstration  Chief Scientist Office of the Scottish Government Health Directorates  Baseline and follow-up 2 | 1,113 participants (560 participants in intervention group, mean age: 45.9 ± 9.0 years; 553 participants in comparison group, mean age: 45.6 ± 8.7 years); 100% male  SES (country-specific income quintiles):  Lower (1^st^ and 2^nd^ quintiles): 252  Higher (3^rd^ to 5^th^ quintiles): 754  *Sub-sample:*  24 UK participants aged 60+ years; 100% male  SES (country-specific income quintiles):  Lower (1^st^ and 2^nd^ quintiles): 3  Higher (3^rd^ to 5^th^ quintiles): 21 | Not stated | Measure: Device-based (activPAL micro accelerometer)  Outcome in review: Continuous |  |  |  |  |  |  |  |  |  | - Body mass index (weight status)  - Upper joint pain limiting activity  - Lower joint pain limiting activity |
| Yen and Hsu, 2020 [78]^†^  Great Britain (England, Scotland, Wales)  International Social Survey Program  2011 Health and Healthcare | Cross-sectional; quantitative non-randomised  Taipei Medical University  2011 Health and Healthcare | 11,250 participants (age range: 65 to 102 years); 10,561 participants with data on active/inactive living; 4,774 male, 5,783 female  *Sub-sample:*  212 participants aged 65+ years in Great Britain; 95 male, 117 female  SES (years of education):  Lower (14 years or less): 196  Higher (more than 14 years): 15 | Health Belief Model | Outcome in review: Dichotomous | ? | ? | ? | + | + |  | ? | + | + | - Smoking (smoking status)  - Alcohol consumption (alcohol consumption)  - Difficulties with activities because of health problems (physical function)  - Bodily aches or pain  - Body mass index (weight status)  - Feel unhappy and depressed (depressive symptoms)  - Loss of confidence in oneself  - Feel you cannot overcome your problems  - General health (perception of general health) |
| Zandieh et al., 2016 [102]^∮^  England | Cross-sectional; mixed-methods study  Erasmus Mundus scholarship supplied by the European Union | 173 participants, quantitative sample (age range: ≥ 65 years; mean age: 74.2 ± 5.9 years); 43% male, 57% female  SES (IMD):  Lower (high-deprivation area): 80  Higher (low-deprivation area): 93 | Not stated | Measure: Device-based (i-gotU GT-600 GPS tracking unit)  Outcome in review: Continuous |  |  |  |  |  |  |  |  |  | - Safety  - Traffic condition  - Pavement condition  - Presence of amenities  - Quietness  - Air quality  - Aesthetics |
| Zandieh, Flacke, et al., 2017 [103]^∮^  England | Cross-sectional; mixed-methods study  Erasmus Mundus scholarship supplied by the European Union | 173 participants, quantitative sample (age range: ≥ 65 years; mean age: 74.2 ± 5.9 years); 43% male, 57% female  SES (IMD):  Lower (high-deprivation area): 80  Higher (low-deprivation area): 93 | Not stated | Measure: Device-based (i-gotU GT-600 GPS tracking unit)  Outcome in review: Continuous |  |  |  |  |  |  |  |  |  | - Land-use mix  - Land-use intensity  - Street connectivity  - Retail density |
| Zandieh, Martinez, et al., 2017 [104]^∮^  England | Cross-sectional; quantitative non-randomised  Erasmus Mundus scholarship supplied by the European Union | 173 participants (age range: ≥ 65 years; mean age: 74.2 ± 5.9 years); 43% male, 57% female  SES (IMD):  Lower (high-deprivation area): 80  Higher (low-deprivation area): 93 | Not stated | Measure: Device-based (i-gotU GT-600 GPS tracking unit)  Outcome in review: Continuous |  |  |  |  |  |  |  |  |  | - Health status |

BRIGHT, Building Research Initiative Group: Chronic Illness Management and Adherence in Transplantation; CARDI, Centre for Ageing Research and Development in Ireland; COVID-19, coronavirus disease; EPAQ2, EPIC Physical Activity Questionnaire; EPIC, European Prospective Investigation into Cancer and Nutrition; EU-WISE, Self-care Support for People with Long Term Conditions, Diabetes and Heart Disease: A Whole System Approach; GPS, Global Positioning System; H2020, Horizon 2020; HADS, Hospital Anxiety and Depression Scale; IMD, Index of Multiple Deprivation; IQ, Intelligence Quotient; NO_2_, nitrogen dioxide; NVQ, National Vocational Qualification; PhD, Doctor of Philosophy; PROTECT, Platform for Research Online to Investigate Genetics and Cognition in Aging; SES, socio-economic status; SIMD, Scottish Index of Multiple Deprivation; SPOTLIGHT, sustainable prevention of obesity through integrated strategies; TSB, Trustee Savings Bank; UK, United Kingdom; V̇O_2_ max, maximal oxygen consumption.

^a^Self-reported physical activity measures were evaluated using criteria adapted from the Quality Assessment of Physical Activity Questionnaires (QAPAQ) checklist [24]. 1: reliability (design and measurement); 2: measurement error; 3: reliability (statistics); 4: face validity; 5: content validity; 6: floor and ceiling effects; 7: construct validity; 8: context validity; 9: purpose validity. Ratings: Yes (+), No (-), or Can’t tell (?). Grey shading indicates the item was not applicable.

^*^Received raw data from original study authors; data extracted by OSM.

^†^Data extracted by OSM (openly available third-party datasets).

^‡^Received summary data from original study authors.

^∮^Data extracted directly from the publication.

^¶^Received syntax file(s) from original study authors; data extracted by OSM (openly available third-party datasets).

**References for openly available datasets accessed, processed, and analysed by OSM (as indicated by a † symbol in Additional file 5):**

| Banks J, Batty GD, Breedvelt J, Coughlin K, Crawford R, Marmot M, et al. English longitudinal study of ageing: waves 0-9, 1998-2019. UK Data Service. 2021. <http://doi.org/10.5255/UKDA-SN-5050-24.> |
| --- |
| Elliott LR, White MP. BlueHealth international survey dataset, 2017-2018. UK Data Service. 2022. <http://doi.org/10.5255/UKDA-SN-8874-2.> |
| European Commission. Eurobarometer 58.2 (Oct-Dec 2002). GESIS Data Archive. 2012. <https://doi.org/10.4232/1.10954>. |
| European Commission. Eurobarometer 88.4 (2017). GESIS Data Archive. 2022. <https://doi.org/10.4232/1.14003>. |
| Gladwell V. Green exercise: the combined effect of the environment and exercise on physical activity and health. UK Data Service. 2016. <http://doi.org/10.5255/UKDA-SN-852253.> |
| ISSP Research Group. International social survey programme: health and health care - ISSP 2011. GESIS Data Archive. 2015. <https://doi.org/10.4232/1.12252>. |
| McNeill A, Vijaykumar S. Conflicting nutrition information. Open Science Framework. 2020. <https://osf.io/zpa5b.> |
| National Centre for Social Research, University College London, Department of Epidemiology and Public Health. Health survey for England, 2003. UK Data Service. 2024. <http://doi.org/10.5255/UKDA-SN-5098-1.> |
| Office for National Statistics, Northern Ireland Statistics and Research Agency. European health interview survey: United Kingdom data, wave 2 and 3, 2013-2020. UK Data Service. 2022. <http://doi.org/10.5255/UKDA-SN-7881-2>.  Office for National Statistics, Social Survey Division. ONS omnibus survey, March 1999. UK Data Service. 2001. <http://doi.org/10.5255/UKDA-SN-4223-1.> |
| Office for National Statistics, Social Survey Division. ONS omnibus survey, September 2000. UK Data Service. 2002. <http://doi.org/10.5255/UKDA-SN-4531-1.> |
| ScotCen Social Research. Scottish health survey, 2014. UK Data Service. 2020. <http://doi.org/10.5255/UKDA-SN-7851-4.> |
| Sport England. Active lives survey, 2016-2017. UK Data Service. 2023. <http://doi.org/10.5255/UKDA-SN-8391-1.> |
| University College London, Department of Epidemiology and Public Health, National Centre for Social Research. Health survey for England, 2006. UK Data Service. 2024. <http://doi.org/10.5255/UKDA-SN-5809-1.> |
| University College London, Department of Epidemiology and Public Health, National Centre for Social Research. Health survey for England, 2007. UK Data Service. 2024. <http://doi.org/10.5255/UKDA-SN-6112-1.> |
