# Supplementary material for: Correlates and determinants of physical activity among older adults of lower versus higher socio-economic status: a systematic review and meta-analysis
Source: Int J Behav Nutr Phys Act. 2025 Jun 23;22:83. doi: 10.1186/s12966-025-01775-y (PMC12183859; doi:10.1186/s12966-025-01775-y)
Supplement: Supplementary file 6 — Additional file 6. Risk of bias of the included studies contributing to each combined meta-analysis. [file 12966_2025_1775_MOESM6_ESM.docx]

**Additional file 6.** Risk of bias of the included studies contributing to each combined meta-analysis

| **Exposures** | **Mixed Methods Appraisal Tool criteria** | | | | | | | | | | | | | | | | | | | | | | | | | | | | | |
| --- | --- | --- | --- | --- | --- | --- | --- | --- | --- | --- | --- | --- | --- | --- | --- | --- | --- | --- | --- | --- | --- | --- | --- | --- | --- | --- | --- | --- | --- | --- |
|  | ***Quantitative randomised controlled trials*** | | | | | | | | | | | | | | | ***Quantitative non-randomised*** | | | | | | | | | | | | | | |
|  | *2.1* | | | *2.2* | | | *2.3* | | | *2.4* | | | *2.5* | | | *3.1* | | | *3.2* | | | *3.3* | | | *3.4* | | | *3.5* | | |
|  | + | – | ? | + | – | ? | + | – | ? | + | – | ? | + | – | ? | + | – | ? | + | – | ? | + | – | ? | + | – | ? | + | – | ? |
| ***Capability*** |  |  |  |  |  |  |  |  |  |  |  |  |  |  |  |  |  |  |  |  |  |  |  |  |  |  |  |  |  |  |
| *A) Physical capability* |  |  |  |  |  |  |  |  |  |  |  |  |  |  |  |  |  |  |  |  |  |  |  |  |  |  |  |  |  |  |
| Physical function  Lower SES (k = 6)  Higher SES (k = 6) |  |  |  |  |  |  |  |  |  |  |  |  |  |  |  | 0  0 | 100  100 | 0  0 | 33  50 | 67  50 | 0  0 | 50  67 | 33  17 | 17  17 | 100  83 | 0  17 | 0  0 | 33  33 | 67  67 | 0  0 |
| *B) Psychological capability* |  |  |  |  |  |  |  |  |  |  |  |  |  |  |  |  |  |  |  |  |  |  |  |  |  |  |  |  |  |  |
| Memory  Lower and higher SES (k = 3) |  |  |  |  |  |  |  |  |  |  |  |  |  |  |  | 33 | 67 | 0 | 0 | 100 | 0 | 67 | 33 | 0 | 67 | 33 | 0 | 0 | 100 | 0 |
| Health literacy  Lower and higher SES (k = 3) |  |  |  |  |  |  |  |  |  |  |  |  |  |  |  | 33 | 67 | 0 | 0 | 100 | 0 | 67 | 33 | 0 | 100 | 0 | 0 | 0 | 100 | 0 |
| ***Opportunity*** |  |  |  |  |  |  |  |  |  |  |  |  |  |  |  |  |  |  |  |  |  |  |  |  |  |  |  |  |  |  |
| *A) Physical opportunity* |  |  |  |  |  |  |  |  |  |  |  |  |  |  |  |  |  |  |  |  |  |  |  |  |  |  |  |  |  |  |
| Amount of green space  Lower and higher SES (k = 3) |  |  |  |  |  |  |  |  |  |  |  |  |  |  |  | 33 | 67 | 0 | 67 | 33 | 0 | 100 | 0 | 0 | 67 | 33 | 0 | 0 | 100 | 0 |
| Built physical activity facilities  Lower and higher SES (k = 4) |  |  |  |  |  |  |  |  |  |  |  |  |  |  |  | 0 | 100 | 0 | 25 | 75 | 0 | 75 | 0 | 25 | 100 | 0 | 0 | 0 | 100 | 0 |
| Natural physical activity facilities  Lower and higher SES (k = 3) |  |  |  |  |  |  |  |  |  |  |  |  |  |  |  | 0 | 100 | 0 | 33 | 67 | 0 | 67 | 0 | 33 | 100 | 0 | 0 | 0 | 100 | 0 |
| Walking and cycling infrastructure  Lower SES (k = 6)  Higher SES (k = 5) |  |  |  |  |  |  |  |  |  |  |  |  |  |  |  | 0  0 | 100  100 | 0  0 | 33  40 | 67  60 | 0  0 | 50  60 | 33  20 | 17  20 | 100  100 | 0  0 | 0  0 | 0  0 | 100  100 | 0  0 |
| *B) Social opportunity* |  |  |  |  |  |  |  |  |  |  |  |  |  |  |  |  |  |  |  |  |  |  |  |  |  |  |  |  |  |  |
| Dog ownership  Lower and higher SES (k = 3) |  |  |  |  |  |  |  |  |  |  |  |  |  |  |  | 33 | 67 | 0 | 33 | 67 | 0 | 100 | 0 | 0 | 100 | 0 | 0 | 0 | 100 | 0 |
| Social participation  Lower SES (k = 5)  Higher SES (k = 4) |  |  |  |  |  |  |  |  |  |  |  |  |  |  |  | 0  0 | 100  100 | 0  0 | 0  0 | 100  100 | 0  0 | 80  75 | 20  25 | 0  0 | 100  100 | 0  0 | 0  0 | 20  25 | 80  75 | 0  0 |
| Social support  Lower and higher SES (k = 3) |  |  |  |  |  |  |  |  |  |  |  |  |  |  |  | 0 | 100 | 0 | 33 | 67 | 0 | 100 | 0 | 0 | 100 | 0 | 0 | 0 | 100 | 0 |
| ***Motivation*** |  |  |  |  |  |  |  |  |  |  |  |  |  |  |  |  |  |  |  |  |  |  |  |  |  |  |  |  |  |  |
| *A) Reflective motivation*  Lower SES (k = 3)^a^ | 100 | 0 | 0 | 100 | 0 | 0 | 0 | 100 | 0 | 100 | 0 | 0 | 100 | 0 | 0 | 0 | 100 | 0 | 0 | 100 | 0 | 50 | 50 | 0 | 50 | 50 | 0 | 0 | 100 | 0 |
| ***Health*** |  |  |  |  |  |  |  |  |  |  |  |  |  |  |  |  |  |  |  |  |  |  |  |  |  |  |  |  |  |  |
| *A) General health and wellbeing* |  |  |  |  |  |  |  |  |  |  |  |  |  |  |  |  |  |  |  |  |  |  |  |  |  |  |  |  |  |  |
| Depressive symptoms  Lower SES (k = 10)  Higher SES (k = 9) |  |  |  |  |  |  |  |  |  |  |  |  |  |  |  | 10  11 | 90  89 | 0  0 | 10  11 | 90  89 | 0  0 | 70  78 | 0  0 | 30  22 | 100  100 | 0  0 | 0  0 | 30  22 | 70  78 | 0  0 |
| Perception of general health  Lower and higher SES (k = 14) |  |  |  |  |  |  |  |  |  |  |  |  |  |  |  | 50 | 50 | 0 | 7 | 93 | 0 | 93 | 0 | 7 | 86 | 14 | 0 | 7 | 93 | 0 |
| Weight status  Lower SES (k = 19)  Higher SES (k = 20)^b^ | 100 | 0 | 0 | 100 | 0 | 0 | 100 | 0 | 0 | 0 | 100 | 0 | 0 | 100 | 0 | 26  26 | 74  74 | 0  0 | 26  32 | 74  68 | 0  0 | 74  79 | 16  11 | 11  11 | 95  89 | 5  11 | 0  0 | 26  26 | 74  74 | 0  0 |
| *B) Health behaviours* |  |  |  |  |  |  |  |  |  |  |  |  |  |  |  |  |  |  |  |  |  |  |  |  |  |  |  |  |  |  |
| Alcohol consumption  Lower and higher SES (k = 7) |  |  |  |  |  |  |  |  |  |  |  |  |  |  |  | 14 | 86 | 0 | 29 | 71 | 0 | 71 | 0 | 29 | 100 | 0 | 0 | 57 | 43 | 0 |
| Fruit and vegetable consumption  Lower and higher SES (k = 3) |  |  |  |  |  |  |  |  |  |  |  |  |  |  |  | 33 | 67 | 0 | 33 | 67 | 0 | 100 | 0 | 0 | 67 | 33 | 0 | 33 | 67 | 0 |
| Sleep  Lower and higher SES (k = 3) |  |  |  |  |  |  |  |  |  |  |  |  |  |  |  | 0 | 100 | 0 | 33 | 67 | 0 | 33 | 0 | 67 | 100 | 0 | 0 | 33 | 67 | 0 |
| Smoking status  Lower SES (k = 8)  Higher SES (k = 9) |  |  |  |  |  |  |  |  |  |  |  |  |  |  |  | 13  11 | 88  89 | 0  0 | 13  22 | 88  78 | 0  0 | 75  67 | 13  11 | 13  22 | 100  100 | 0  0 | 0  0 | 38  44 | 63  56 | 0  0 |

*Note:* Risk of bias was assessed using the Mixed Methods Appraisal Tool, Version 2018. SES, socio-economic status; k, number of studies. “Yes” rating (+): the criterion is met; “No” rating (–): the criterion is not met; “Can’t tell” rating (?): there is not enough information to judge if the criterion is met or not. Criterion 2.1: Is randomisation appropriately performed? Criterion 2.2: Are the groups comparable at baseline? Criterion 2.3: Are there complete outcome data? Criterion 2.4: Are outcome assessors blinded to the intervention provided? Criterion 2.5: Did the participants adhere to the assigned intervention? Criterion 3.1: Are the participants representative of the target population? Criterion 3.2: Are measurements appropriate regarding both the outcome and intervention (or exposure)? Criterion 3.3: Are there complete outcome data? Criterion 3.4: Are the confounders accounted for in the design and analysis? Criterion 3.5: During the study period, is the intervention administered (or the exposure occurred) as intended?

^a^One quantitative randomised controlled trial and two quantitative non-randomised studies.

^b^One quantitative randomised controlled trial and nineteen quantitative non-randomised studies.
